# Supplementary material for: The temporal variation in pesticide concentrations within matured French wines
Source: PLoS One. 2025 Feb 11;20(2):e0317086. doi: 10.1371/journal.pone.0317086 (PMC11813125; doi:10.1371/journal.pone.0317086)
Supplement: S1 Table — (DOCX) [file pone.0317086.s001.docx]

**Table S1 A list of tested French wines, including sample codes, growing regions, and years of production**

| **Sample**  **name** | **Region (AOC)** | **Year** | **Additional info on the bottle (including winery)** |
| --- | --- | --- | --- |
| F1 | Bourgogne | 1974 | Pommard - Emile Chandesais |
| F2 | Bordeaux | 1973 | Bordeaux Rouge |
| F3 | Côtes du Rhône  (Châteauneuf-du-Pape) | 1980 | Châteauneuf-du-Pape |
| F4 | Bordeaux (Pomerol) | 1959 | Château Beauregard |
| F5 | Côtes du Rhône  (Châteauneuf-du-Pape) | 1970 | Domaine des Sechau |
| F6 | Côtes du Rhône | 1972 | Castée du Roy |
| F7 | Bordeaux (Saint-Emilion) | 1975 | Lussac saint-Emilion |
| F8 | Bordeaux | 1973 | Clos du Roy |
| F9 | Bordeaux (Cessac) | 1987 | Haut Cessac |
| F10 | Bourgogne | 1993 | Bourgogne Passetoutgrains |
| F11 | Bordeaux (Médoc) | 1937 | Château Angludet, Cantenac-Margaux |
| F12 | Bordeaux (Médoc) | 1964 | Château Buisson |
| F13 | Bordeaux (Graves) | 1996 | Château Coucheroy |
| F14 | Côtes du Rhône | 1998 | Château Signac |
| F15 | Loire (Bourgueil) | 1979 |  |
| F16 | Bourgogne | 1978 | Vosne Romanée - Marcel Bocquenet |
| F17 | Côtes du Rhône | 1998 | Château du Vieux Tinel |
| F18 | Bordeaux (Gironde) | 1982 | Grand Cru Classé Saint-Emilion |
| F19 | Bordeaux | 1970 | Château La fleur canon - cotes canon fronsac |
| F20 | Côtes du Rhône | 1998 | Domaine Saint Gayan |
| F21 | Bourgogne | 1990 | Vosne Romanée - Jean pierre Mugneret |
| F22 | Côtes du Rhône  (Châteauneuf-du-Pape) | 2000 | Domaine des Relagne |
| F23 | Bordeaux (Gironde) | 1973 | Grand Vin Côtes de Fronsac - Grandys |
| F24 | Bordeaux (Libournais) | 1990 | Château Pontet-Clauzure |
| F25 | Bordeaux (Médoc) | 1993 | Château la Tour l'Aspic |
| F26 | Côtes du Rhône | 1964 | E. Veyrat - Reserve des Eveques d'Orange - Gigondas |
| F27 | Bordeaux (Saint-Emilion) | 1937 | Château Franc-Pourret, Saint-Emilion |
| F28 | Bordeaux | 1967 | Château Guiteronde - bordeaux supérieur |
| F29 | Bordeaux (Médoc) | 1969 | Château Bries-Caillou |
| F30 | Côtes du Rhône | 1978 | Châteauneuf du pape - R. Chassaing |
| F31 | Bourgogne | 1977 | Chambolle musigny - leymarie |
| F32 | Bordeaux (Saint-Emilion) | 1982 | Château cap de mourlin |
| F33 |  | s 60 | Vin du patron - C.E.E. Sélectionné |
| F34 | Bordeaux | 1959 | Château Haut Bages Monpelou |
| F35 | Bordeaux | 1996 | Château Puyanché |
| F36 | Côtes du Rhône  (Coteaux du Tricastin) | 1997 |  |
| F37 | Loire (Chinon) | 1997 | Domaine de la Noblaie |
| F38 | Bourgogne (Vosne-romanée) | 1976 |  |
| F39 | Bourgogne (Corton) | 1983 | Château Couvent des Cordeliers |
| F40 | Bordeaux (Margaux) | 1989 | Château Laroque |
| F41 | Languedoc-Roussillon | 1986 | Les Vignobles des Côtes d'Agly, Vin De Pays Du Val D'Agly |
| F42 | Languedoc-Roussillon  (Minervois La Livinière) | 1998 | La Chapelle de Calamiac |
| F43 | Côtes du Rhône | 1999 | Domaine du Grand Vaucroze |
| F44 | Côtes du Rhône | 1987 | Domaine Boisson |
| F45 | Côtes du Rhône (Vacqueyras) | 1995 | Domaine Chantegut |
| F46 | Bordeaux (Côtes De Blaye) | 1996 | Château De La Salle |
| F47 | Bordeaux | 1996 | Château Victoria |
| F48 | Bourgueil | 1999 | Domaine Guion |
| F49 | Beaujolais | 1971 |  |
| F50 | Bordeaux | 1986 | Château Garrousat |
| F51 | Bordeaux | 1993 | Chateau Labegorce margaux |
| F52 | Bordeaux | 1998 | Chateau La Cabanne - pomerol |
| F53 | Langduedoc | 1998 | Chateau mauvezin |
| F54 | Bordeaux | 1986 | Chateau Romas |
| F55 | - | - | Broken |
| F56 | Bordeaux | 1995 | Chateau peyreau |
| F57 | Bordeaux (Saint-Emilion) | 1999 | Chateau le gay |
| F58 | Bordeaux (Gironde) | 1995 | Chateau lalande |
| F59 | Bordeaux | 1999 | Chateau chollet |
| F60 | Bordeaux | 1999 | Chateaux Cantenac Brown/Margaux |
| F61 | Gironde/ Bordeaux | 1997 | Château l'érmitage R, Thomas/haut Médoc |
| F62 | Médoc/ Bordeaux | 1970 | Château Monbousquet/Saint Emilion |
| F63 | Saint Emilion | 1975 | Château Giraudon/ |
| F64 | Bergerac | 1989 | Château la clotte/ |
| F65 | Puissequin Saint Emilion | 1978 | Chateau les hautes graves/Pomerol |
| F66 | Bordeaux | 1973 | Chateau Puy Blanquet |
| F67 | Rhone | 1999 | Château Moulin de Lavaud/pomerol |
| F68 | Saint Emilion | 1971 | Château Le Prieure |
| F69 | Bordeaux | 1975 | Grand Pey Lescours |
| F70 | Bourgongne | 2000 | Chaâteau de Lescours |
| F71 | Saint Emilion | 1982 | Château cheval blanc |
| F72 | Saome et loire | 1969 | Chateau Lagrange |
| F73 | Saint Emilion | 1969 | Château pontet canet |
| F74 | Rhône | 1983 | Château Pavie Macquin |
| F75 | Saint Emilion | 1970 | Château Le Pey |
| F76 | -Label is not readable- | 1964 | Château Lociando -Mallet |
| F77 | Saint Emilion | 1993 | Château La Gurgue |
| F78 | Gironde/Bordeaux | 1983 | Château Laroze |
| F79 | Pauillac (Gironde) | 1970 | Château Roc De Cambes |
| F80 | Saint Emilion | 1964 | Chateau Labegorce margaux |
| F81 | Médoc/Bordeaux | 1994 | Chateau La Cabanne - pomerol |
| F82 | Haut-Médoc / Gironde / Bordeaux | 1990 | Chateau mauvezin |
| F83 | Margaux/irone/Bordeaux | 1991 | Chateau Romas |
| F84 | Saint Emilion | 1990 | Chateau peyreau |
| F85 | Côtes De Bourg/Bordeaux | 1981 | Chateau le gay |

* The exact composition of grape varieties for these samples could not be found. The composition given is the one most commonly found in the region
