# Supplementary material for: The temporal variation in pesticide concentrations within matured French wines
Source: PLoS One. 2025 Feb 11;20(2):e0317086. doi: 10.1371/journal.pone.0317086 (PMC11813125; doi:10.1371/journal.pone.0317086)
Supplement: S2 Table — (DOCX) [file pone.0317086.s002.docx]

**Table S2 Percentage of recovery for active ingredients for wine extraction**

| **Active ingredient** | **Recovery (%)** | **Active ingredient** | **Recovery (%)** |
| --- | --- | --- | --- |
| Azoxystrobin | 53.5 | Metalaxyl | 65.3 |
| Benalaxyl | 73.2 | Piperonyl butoxide | 118.0 |
| Cadusafos | 53.2 | Prochloraz | 33.8 |
| Carbaryl | 21.3 | Propanil | 46.7 |
| Carbendazim | 100.0* | Pyrimethanil | 36.5 |
| Chlorpropham | 229.1 | Spinosad A | 21.1 |
| Diethofencarb | 46.3 | Spinosad D | 25.0 |
| Difenoconazole | 44.0 | Tebuconazole | 105.7 |
| Dimethomorph | 71.3 | Tebufenozide | 77.3 |
| Diuron | 39.7 | Triadimefon | 69.7 |
| Fenbuconazole | 50.7 | Triadimenol | 136.0 |
| Hexaconazole | 72.0 |  |  |

* For Carbendazim. a separate standard series/calibration curve was prepared and adapted to the matrix due to the poor efficiency of the standard method

** For all LCMSMS components: LOD = 0.0003mg/L. LOQ = 0.001mg/L
