# Supplementary material for: The temporal variation in pesticide concentrations within matured French wines
Source: PLoS One. 2025 Feb 11;20(2):e0317086. doi: 10.1371/journal.pone.0317086 (PMC11813125; doi:10.1371/journal.pone.0317086)
Supplement: S3 Table — (DOCX) [file pone.0317086.s003.docx]

**Table S3 Percentage recovery of active ingredients for wine sediment extraction**

| **Active ingredient** | **Recovery (%)** | **Active ingredient** | **Recovery (%)** |
| --- | --- | --- | --- |
| Atrazine | 40.4 | Difenoconazole | 66.4 |
| Azoxystrobine | 87.9 | Dimethomorph | 79.3 |
| Benalaxyl | 68.5 | Metalaxyl | 108.0 |
| Cadusafos | 63.4 | Pyrimethanil | 71.5 |
| Carbaryl | 83.4 | Tebuconazole | 94.2 |
| Carbendazim | 39.2 | Tebufenozide | 106.0 |
| Diethofencarb | 89.2 |  |  |
