# Supplementary material for: The temporal variation in pesticide concentrations within matured French wines
Source: PLoS One. 2025 Feb 11;20(2):e0317086. doi: 10.1371/journal.pone.0317086 (PMC11813125; doi:10.1371/journal.pone.0317086)
Supplement: S4 Table — Column 2 -the percentage of samples with concentrations higher than LOQ. Column 3—the average concentration of each pesticide across all samples with levels above the LOQ excluding the zero samples. Column 4 –ADI values. Column 5—the release year for each pesticide, respectively. (DOCX) [file pone.0317086.s004.docx]

**Table S4 A summary of different pesticides detected in wine, with concentrations exceeding the LOQ. Column 2 -the percentage of samples with concentrations higher than LOQ. Column 3 - the average concentration of each pesticide across all samples with levels above the LOQ excluding the zero samples. Column 4 – ADI values. Column 5 - the release year for each pesticide, respectively**

| **Pesticide** | **% positive samples**  **(n = 84)** | **The mean concentration of positive samples >LOQ**  **(mg/L wine)** | **ADI**  **(mg/kgBW/d)** | **Introduction year PPP on the market** |
| --- | --- | --- | --- | --- |
| Azoxystrobin | 6% | 3.00E-04 | 0.2 | 1992 |
| Benalaxyl | 14% | 1.71E-04 | 0.04 | 1981 |
| Carbaryl | 24% | 2.48E-01 | 0.0075 | ~ 1957 |
| Carbendazim | 88% | 3.78E-03 | 0.02 | 1973 |
| Chlorpropham | 4% | 2.56E-04 | 0.05 | 1951 |
| Diethofencarb | 18% | 1.08E-03 | 0.43 | 1986 |
| Difenoconazole | 13% | 1.42E-03 | 0.01 | 1988 |
| Dimethomorph | 32% | 2.09E-03 | 0.05 | 1988 |
| Diuron | 13% | 1.84E-04 | 0.007 | ~ 1951 |
| Fenbuconazole | 1% | 9.08E-05 | 0.006 | 1992 |
| Hexaconazole | 11% | 3.99E-04 | 0.005 | 1986 |
| Metalaxyl | 40% | 1.39E-03 | 0.08 | 1977 |
| Piperonyl butoxide | 43% | 5.17E-05 |  | ~ 1950 |
| Prochloraz | 1% | 1.63E-02 | 0.01 | 1977 |
| Propanil | 7% | 1.4E-04 | 0.02 | 1960 |
| Pyrimethanil | 26% | 1.31E-03 | 0.17 | 1998 |
| Tebuconazole | 18% | 9.15E-04 | 0.03 | 1986 |
| Tebufenozide | 14% | 1.16E-02 | 0.02 | 1994 |
| Triadimefon | 11% | 1.26E-04 | 0.03 | 1976 |
| Triadimenol | 12% | 4.76E-04 | 0.05 | 1978 |
