# Supplementary material for: The temporal variation in pesticide concentrations within matured French wines
Source: PLoS One. 2025 Feb 11;20(2):e0317086. doi: 10.1371/journal.pone.0317086 (PMC11813125; doi:10.1371/journal.pone.0317086)
Supplement: S5 Table — Column 2—the type of PPP. Column 3- the main target organism for each pesticide. Column 4 -the mode of action for each PPP (systemic and contact). Column 5—the MRL for grapes as per the latest European Commission regulations. Column 6—indicates whether the pesticide is authorized for use in the European Union as of 2022, according to the European Commission laws [37]. (DOCX) [file pone.0317086.s005.docx]

**Table S5 Pesticides detected in wine (1st column). Column 2 - the type of PPP. Column 3- the main target organism for each pesticide. Column 4 -the mode of action for each PPP (systemic and contact). Column 5 - the MRL for grapes as per the latest European Commission regulations. Column 6 - indicates whether the pesticide is authorized for use in the European Union as of 2022, according to the European Commission laws [37]**

| Name | Type | Target organism | Function | MRL^(1)^  *(mg/kg)* | Approval  (2022) | Sources |
| --- | --- | --- | --- | --- | --- | --- |
| Azoxystrobin | Fungicide | Rusting. downy mildew. black rot | Systemic | 3 | Approved | [38]  [39] |
| Benalaxyl | Fungicide | Downy mildew | Systemic | 0.3 | Not approved | [40]  [39] |
| Cadusafos | Insecticide.  nematocide | Nematodes | Contact | 0.01* | Not approved | [41]  [42] |
| Carbaryl | Insecticide | Aphids. butterflies | Contact | 0.01* | Not approved | [43]  [42] |
| Carbendazim | Fungicide | Gray mold | Systemic | 0.5 | Not approved | [44]  [39] |
| Chlorpropham | Herbicide | Mono- and dicotyls | Systemic | 0.01* | Not approved | [45]  [46] |
| Diethofencarb | Fungicide | Gray mold | Systemic | 0.9 | Not approved | [47]  [46] |
| Difenoconazole | Fungicide | Leaf spot pathogens | Systemic | 3 | Approved | [41]  [48] |
| Dimethomorph | Fungicide | Downy mildew | Systemic | 3 | Approved | [49]  [50]  [39] |
| Diuron | Herbicide | Mono- and dicotyls | Systemic | 0.01* | Not approved | [41]  [46] |
| Fenbuconazole | Fungicide | Powdery mildew. black rot | Systemic | 1.5 | Not approved | [51]  [46] |
| Hexaconazole | Fungicide | Powdery mildew. rusting | Systemic | 0.01* | Not approved | [41]  [46] |
| Metalaxyl | Fungicide | Mildew | Systemic | 1 | Approved | [52]  [46] |
| Piperonyl-butoxide | Synergist in insecticides |  |  |  | (2) | [53] |
| Prochloraz | Fungicide | Anthracnose causative agents | Contact | 0.03** | Not approved | [54] |
| Propanil | Herbicide | Mono- and dicotyls | Contact | 0.01* | Not approved | [55]  [46] |
| Pyrimethanil | Fungicide | Gray mold | Locally Systemic | 5 | Approved | [41]  [56] |

| Name | Type | Target organism | Function | MRL^(1)^  (mg/kg) | Approval  (2022) | Sources |
| --- | --- | --- | --- | --- | --- | --- |
| Spinosad A  Spinosad D | Insecticide | Tripsen | Contact | 0.5 | Approved | [57]  [42] |
| Tebuconazole | Fungicide | Powdery mildew | Systemic | 1 | Approved | [43]  [46] |
| Tebufenozide | Insecticide | *Paralobesia viteana* | Contact | 4 | Approved | [43]  [42] |
| Triadimefon | Fungicide | Powdery mildew | Systemic | 0.01* | Not approved | [40]  [46] |
| Triadimenol | Fungicide | Powdery mildew. rusting | Systemic | 0.3 | Not approved | [58]  [46] |

(1) MRL always refers to raw products, not finished products. In this case to wine grapes and not wine.

(2) The approval of the substance Piperonyl butoxide has expired and has not yet been re-evaluated in the EU in the framework of 91/414/EEC.

* Article 18.1(b) of the regulation (EC) No. 396/2005 states that for products without a specifically described MRL. a default value of 0.01 mg/kg is assigned.

** The MRL is chosen according to the minimum quantifiable concentration (LOQ). For more info: see 8.4.1.2. LOQ

**References**

1. AgChemAccess. (2021a). Azoxystrobin. [http://www.agchemaccess.com/Azoxystrobin#:%7E:text=The%20mode%20of%20action%20of.Complex%20III%20within%20the%20mitochondrion](http://www.agchemaccess.com/Azoxystrobin%23:%7E:text=The%20mode%20of%20action%20of.Complex%20III%20within%20the%20mitochondrion%20) (accessed on 26 April 2023)
2. Fungicide resistance action committee. (2021). FRAC Classification of Fungicides [Poster]. FRAC. [https://www.corder.be/sites/default/files/2022-04/frac-moa-poster-2021.pdf](https://www.corder.be/sites/default/files/2022-04/frac-moa-poster-2021.pdf%20) (accessed on 14 May 2023)
3. S. Navarro. A. Barba. G. Navarro. N. Vela. and J. Oliva. “Multiresidue method for the rapid determination – in grape. must and wine – of fungicides frequently used on vineyards.” *J Chromatogr A*. vol. 882. no. 1. pp. 221–229. 2000. doi: https://doi.org/10.1016/S0021-9673(00)00337-X.
4. K. A. Lewis. J. Tzilivakis. D. J. Warner. and A. Green. “An international database for pesticide risk assessments and management.” Human and Ecological Risk Assessment: An International Journal. vol. 22. no. 4. pp. 1050–1064. May 2016. doi: 10.1080/10807039.2015.1133242
5. Insecticide Resistance Action Committee. (2022. March). IRAC Mode of action classification scheme (Versie 10.2). [https://irac-online.org/documents/moa-classification/](https://irac-online.org/documents/moa-classification/%20) (accessed on 8 March 2023)
6. C. Bond. A. Cross. K. Buhl. and D. Stone. (2016). Carbaryl General Fact Sheet. National GBM Information Center. [http://npic.orst.edu/factsheets/carbarylgen.html](http://npic.orst.edu/factsheets/carbarylgen.html%20) (accessed on 6 April 2023)
7. F. Eissa. A. A. Helalia. M. A. Khorshed. and M. A. El-Sisi. “Monitoring of multi-class pesticide residues in green grape and their potential risk for Egyptian consumer.” Nature and Science Journal. vol. 11. pp. 110–115. Nov. 2013
8. United States Environmental Protection Agency. (1996). R.E.D. FACTS: Chlorpropham. [https://archive.epa.gov/pesticides/reregistration/web/pdf/0271fact.pdf](https://archive.epa.gov/pesticides/reregistration/web/pdf/0271fact.pdf%20) (accessed on 17/09/2023)
9. Herbicide Resistance Action Committee. (2022). HRAC Mode of Action Classification 2022 Map. HRAC.. from [https://hracglobal.com/tools/hrac-mode-of-action-classification-2022-map](https://hracglobal.com/tools/hrac-mode-of-action-classification-2022-map%20) (accessed on 10 March 2023)
10. P. Cabras and E. Conte. “Pesticide residues in grapes and wine in Italy.” Food Addit Contam. vol. 18. no. 10. pp. 880–885. Oct. 2001. doi: 10.1080/02652030120491
11. AgChemAccess. (2021b). Difenoconazole. [http://www.agchemaccess.com/Difenoconazole](http://www.agchemaccess.com/Difenoconazole%20) (accessed on 26 April 2023)
12. G. T. Bakırcı. D. B. Yaman Acay. F. Bakırcı. and S. Ötleş. “Pesticide residues in fruits and vegetables from the Aegean region. Turkey.” Food Chem. vol. 160. pp. 379–392. Oct. 2014. doi: 10.1016/j.foodchem.2014.02.051.
13. X. Xu et al.. “Distribution and migration study of pesticides between peel and pulp in grape by online gel permeation chromatography–gas chromatography/mass spectrometry.” Food Chem. vol. 135. no. 1. pp. 161–169. 2012. doi: https://doi.org/10.1016/j.foodchem.2012.04.052.
14. V. Jankuloska. I. Karov. and G. Pavlovska. “Determination of pesticide residues (insecticides) in apples and dietary risk assessment.” vol. 6. pp. 96–101. Sep. 2023.
15. G. N. AGRIOS. “Chapter nine - Control of plant diseases.” in Plant Pathology (Fifth Edition). G. N. Agrios. Ed.. San Diego: Academic Press. 2005. pp. 293–353. doi: https://doi.org/10.1016/B978-0-08-047378-9.50015-4.
16. Cross. A.. Bond. C.. Buhl. K.. & Jenkins. J. (2017). Piperonyl Butoxide General Fact Sheet. National GBM Information Center. [http://npic.orst.edu/factsheets/pbogen.html](http://npic.orst.edu/factsheets/pbogen.html%20) (accessed on 6 April 2022)
17. Hayes’ Handbook of Pesticide Toxicology. Elsevier. (2010). doi: 10.1016/C2009-1-03818-0.
18. J. Liu. (2014). Propanil. In P. Wexler (Red.). Encyclopedia of Toxicology (3rd edition. pp. 1092–1093). Academic Press.
19. AgChemAccess. (2021c). Pyrimethanil. [http://www.agchemaccess.com/Pyrimethanil](http://www.agchemaccess.com/Pyrimethanil%20) (accessed on 26 April 2023)
20. T.R. Bunch. C. Bond. K. Buhl. and D. Stone. (2014). “Spinosad General Fact Sheet. National GBM Information Center”. [http://npic.orst.edu/factsheets/spinosadgen.html](http://npic.orst.edu/factsheets/spinosadgen.html%20) (accessed on 6 April 2023)
21. U.S. Environmental Protection Agency. (2006. August). Triadimefon Reregistration Eligibility Decision (RED) and Triadimenol Tolerance Reassessment and Risk Management Decision (TRED) Fact Sheet. [https://archive.epa.gov/pesticides/reregistration/web/pdf/triadimefon_red.pdf](https://archive.epa.gov/pesticides/reregistration/web/pdf/triadimefon_red.pdf%20) (accessed on 09/10/2023)
