# Supplementary material for: The temporal variation in pesticide concentrations within matured French wines
Source: PLoS One. 2025 Feb 11;20(2):e0317086. doi: 10.1371/journal.pone.0317086 (PMC11813125; doi:10.1371/journal.pone.0317086)
Supplement: S6 Table — (DOCX) [file pone.0317086.s006.docx]

**Table S6 The residues (mg/L) of pesticides detected liquid phase in wine samples (in green values above 0.01mg/L, in yellow values above 0,05mg/L)**

| **Sample number** | **Azoxystrobine** | **Benalaxyl** | **Cadusafos** | **Carbaryl** | **Carbendazim** | **Chlorpropham** | **Diethofencarb** | **Difenoconazole** | **Dimethomorph** | **Diuron** | **Fenbuconazole** |
| --- | --- | --- | --- | --- | --- | --- | --- | --- | --- | --- | --- |
| **EU allowed content [mg/L]** | 3 | 0,3 |  | 0,01 | 0,5 | 0,01 | 0,01 | 3 | 3 | 0,01 | 1,5 |
| **F01** | <LOD | <LOD | <LOD | <LOD | <LOD | 0,00015 | <LOD | 0,00247 | <LOD | <LOD | <LOD |
| **F02** | <LOD | <LOD | <LOQ | 0,00043 | 0,00101 | <LOD | <LOD | <LOD | <LOD | <LOD | <LOD |
| **F03** | <LOD | <LOD | <LOD | <LOD | 0,01026 | 0,00014 | <LOD | <LOD | <LOD | <LOD | <LOD |
| **F04** | <LOD | <LOD | <LOQ | <LOD | <LOD | <LOD | <LOD | <LOD | <LOD | <LOD | <LOD |
| **F05** | <LOD | <LOD | <LOD | 0,01320 | 0,00169 | <LOD | <LOD | <LOD | <LOD | <LOD | <LOD |
| **F06** | <LOD | <LOD | <LOD | 0,00224 | 0,01160 | <LOD | <LOD | <LOD | <LOD | <LOD | <LOD |
| **F07** | <LOD | <LOD | <LOQ | 0,00032 | 0,00198 | <LOD | <LOD | <LOD | <LOD | <LOD | <LOD |
| **F08** | <LOD | <LOD | <LOD | <LOD | 0,00011 | <LOD | <LOD | <LOD | <LOD | <LOD | <LOD |
| **F09** | <LOD | 0,00009 | <LOQ | <LOD | 0,00053 | <LOD | <LOD | <LOD | <LOD | <LOD | <LOD |
| **F10** | <LOD | <LOQ | <LOD | <LOD | 0,00056 | <LOD | <LOD | <LOD | 0,00009 | <LOD | <LOD |
| **F11** | <LOD | <LOD | <LOQ | <LOD | <LOD | <LOD | <LOD | <LOD | <LOD | <LOD | <LOD |
| **F12** | <LOD | <LOD | <LOQ | <LOD | <LOD | <LOD | <LOD | <LOD | <LOD | <LOD | <LOD |
| **F13** | <LOD | <LOD | <LOD | <LOD | 0,03480 | <LOD | 0,00266 | <LOD | 0,00038 | 0,00011 | <LOD |
| **F14** | <LOD | 0,00007 | <LOD | <LOD | 0,00025 | 0,00047 | <LOD | <LOD | <LOQ | <LOQ | <LOD |
| **F15** | <LOD | <LOD | <LOD | <LOD | 0,00043 | <LOD | <LOD | <LOD | <LOD | <LOD | <LOD |
| **F16** | <LOD | <LOD | <LOQ | 0,00075 | 0,00051 | <LOD | <LOD | <LOD | <LOD | <LOD | <LOD |
| **F17** | <LOD | <LOD | <LOQ | <LOD | 0,00020 | <LOD | <LOD | <LOQ | 0,00006 | 0,00029 | <LOD |
| **F18** | <LOD | <LOD | <LOQ | <LOD | 0,00015 | <LOD | <LOD | <LOD | <LOD | <LOD | <LOD |
| **F19** | <LOD | <LOD | <LOQ | <LOD | 0,00008 | <LOD | <LOD | <LOD | <LOD | <LOD | <LOD |
| **F20** | 0,00008 | <LOD | <LOQ | <LOD | <LOD | <LOD | <LOD | <LOD | 0,00009 | <LOD | <LOD |
| **F21** | <LOD | 0,00006 | <LOQ | <LOD | 0,00288 | <LOD | 0,00022 | <LOD | <LOD | <LOD | <LOD |
| **F22** | <LOQ | <LOD | <LOQ | <LOD | 0,00012 | <LOD | <LOD | <LOQ | 0,01230 | 0,00021 | <LOD |
| **F23** | <LOD | <LOD | <LOD | 0,00140 | 0,00419 | <LOD | <LOD | <LOD | <LOQ | <LOD | <LOD |
| **F24** | <LOD | <LOD | <LOQ | <LOD | 0,01800 | <LOD | <LOD | <LOD | <LOD | 0,00016 | <LOD |
| **F25** | <LOD | <LOD | <LOQ | <LOD | 0,01361 | <LOD | 0,00037 | <LOD | 0,00009 | <LOD | <LOD |
| **F26** | <LOD | <LOD | <LOQ | <LOD | 0,00007 | <LOD | <LOD | <LOD | <LOD | <LOD | <LOD |
| **F27** | <LOD | <LOD | <LOQ | <LOD | <LOD | <LOD | <LOD | <LOD | <LOD | <LOD | <LOD |
| **F28** | <LOD | <LOD | <LOQ | <LOD | 0,00006 | <LOD | <LOD | <LOD | <LOD | <LOD | <LOD |
| **F29** | <LOD | <LOD | <LOQ | 2,95072 | 0,00009 | <LOD | <LOD | <LOD | <LOD | <LOD | <LOD |
| **F30** | <LOD | <LOD | <LOQ | 0,00086 | 0,00125 | <LOD | <LOD | <LOD | <LOD | <LOD | <LOD |
| **F31** | <LOD | <LOD | <LOD | 0,00181 | 0,00069 | <LOD | <LOD | 0,00038 | <LOD | <LOD | <LOD |
| **F32** | <LOD | <LOD | <LOQ | <LOQ | 0,00491 | <LOD | <LOD | <LOQ | <LOD | <LOD | <LOD |
| **F33** | <LOD | <LOQ | <LOD | 0,00485 | 0,00652 | <LOD | <LOD | <LOD | <LOD | <LOD | <LOD |
| **F34** | <LOD | <LOD | <LOQ | <LOD | <LOD | <LOD | <LOD | <LOD | <LOD | <LOD | <LOD |
| **F35** | <LOD | <LOD | <LOD | <LOD | 0,00060 | <LOD | <LOD | <LOD | 0,00008 | 0,00021 | <LOD |
| **F36** | <LOD | 0,00006 | <LOQ | <LOD | 0,00991 | <LOD | <LOD | <LOD | 0,00364 | 0,00018 | <LOD |
| **F37** | <LOD | 0,00006 | <LOD | <LOD | 0,00012 | <LOD | <LOD | <LOD | <LOD | <LOD | <LOD |
| **F38** | <LOD | <LOD | <LOQ | 0,00080 | 0,00120 | <LOD | <LOD | <LOD | <LOD | <LOD | <LOD |
| **F39** | <LOD | <LOD | <LOQ | <LOD | 0,00083 | <LOD | <LOD | <LOD | <LOD | <LOD | <LOD |
| **F40** | <LOD | <LOD | <LOQ | <LOD | 0,00017 | <LOD | <LOD | <LOD | <LOD | <LOD | <LOD |
| **F41** | <LOD | <LOD | <LOQ | <LOD | 0,00025 | <LOD | <LOD | <LOD | <LOD | <LOD | <LOD |
| **F42** | <LOD | <LOD | <LOD | <LOD | 0,00016 | <LOD | <LOD | <LOQ | <LOD | <LOD | <LOD |
| **F43** | 0,00053 | <LOQ | <LOD | <LOD | <LOD | <LOD | <LOD | <LOD | 0,00016 | <LOQ | <LOD |
| **F44** | <LOD | <LOD | <LOQ | <LOD | 0,00021 | <LOD | <LOD | <LOD | <LOD | <LOD | <LOD |
| **F45** | <LOD | 0,00056 | <LOQ | <LOD | 0,00114 | <LOD | <LOD | <LOD | 0,00018 | 0,00012 | <LOD |
| **F46** | <LOD | <LOD | <LOQ | <LOD | 0,01869 | <LOD | <LOD | <LOQ | 0,00069 | <LOQ | 0,00009 |
| **F47** | <LOD | <LOD | <LOQ | <LOD | 0,00054 | <LOD | <LOQ | <LOD | 0,00737 | <LOQ | <LOD |
| **F48** | <LOD | <LOD | <LOQ | <LOD | 0,00059 | <LOD | <LOD | <LOD | <LOQ | <LOD | <LOD |
| **F49** | <LOD | <LOD | <LOQ | 0,00441 | 0,00684 | <LOD | <LOD | <LOD | <LOD | <LOD | <LOD |
| **F50** | <LOD | 0,00029 | <LOQ | <LOD | 0,00115 | <LOD | <LOQ | <LOD | <LOD | <LOD | <LOD |
| **F51** | <LOD | <LOD | <LOQ | <LOD | 0,00056 | <LOD | 0,00041 | <LOQ | 0,00035 | <LOD | <LOD |
| **F52** | <LOD | <LOD | <LOD | <LOD | 0,00209 | <LOD | 0,00388 | <LOD | 0,00007 | <LOD | <LOD |
| **F53** | <LOD | <LOD | <LOQ | <LOD | 0,00142 | <LOD | <LOD | <LOD | <LOD | <LOD | <LOD |
| **F54** | <LOD | <LOQ | <LOQ | <LOD | 0,00029 | <LOD | <LOD | <LOD | <LOD | <LOD | <LOD |
| **F56** | <LOQ | <LOQ | <LOQ | <LOD | <LOQ | <LOD | 0,00023 | <LOQ | 0,00210 | <LOD | <LOD |
| **F57** | <LOD | <LOD | <LOD | <LOD | 0,00009 | <LOD | <LOQ | <LOD | <LOQ | <LOD | <LOD |
| **F58** | <LOD | <LOD | <LOQ | <LOD | 0,00026 | <LOD | <LOD | <LOD | 0,00007 | <LOD | <LOD |
| **F59** | <LOD | <LOD | <LOQ | <LOD | 0,00082 | <LOD | 0,00107 | <LOD | <LOQ | <LOD | <LOD |
| **F60** | 0,01137 | <LOD | <LOD | 0,00140 | 0,01308 | <LOD | 0,00794 | <LOD | <LOQ | <LOD | <LOD |
| **F61** | <LOD | <LOD | <LOD | <LOD | 0,00120 | <LOD | 0,00160 | <LOQ | 0,00009 | <LOD | <LOD |
| **F62** | <LOD | <LOD | <LOQ | <LOD | 0,00023 | <LOD | <LOD | <LOD | <LOD | <LOD | <LOD |
| **F63** | <LOD | <LOD | <LOQ | <LOQ | 0,00419 | <LOD | <LOD | <LOD | <LOD | <LOD | <LOD |
| **F64** | <LOD | <LOD | <LOQ | <LOD | 0,00032 | <LOD | <LOD | <LOD | <LOD | <LOD | <LOD |
| **F65** | <LOD | <LOD | <LOQ | 0,00964 | 0,00108 | <LOD | <LOD | <LOD | <LOD | <LOD | <LOD |
| **F66** | <LOD | <LOD | <LOD | 0,00021 | 0,00118 | <LOD | <LOD | <LOD | <LOD | <LOD | <LOD |
| **F67** | <LOD | <LOD | <LOD | <LOD | 0,00053 | <LOD | <LOD | <LOD | 0,00009 | <LOD | <LOD |
| **F68** | <LOD | <LOD | <LOQ | 0,01690 | <LOD | <LOD | <LOD | <LOD | <LOD | <LOD | <LOD |
| **F69** | <LOD | <LOD | <LOD | 0,00023 | 0,01738 | <LOD | <LOD | <LOD | <LOD | <LOD | <LOD |
| **F70** | <LOD | <LOD | <LOD | <LOD | 0,00413 | <LOD | 0,00068 | <LOD | <LOQ | <LOD | <LOD |
| **F71** | <LOD | <LOD | <LOD | <LOD | 0,00058 | <LOD | <LOD | <LOD | <LOD | <LOD | <LOD |
| **F72** | <LOD | <LOD | <LOD | 0,00087 | 0,00054 | <LOD | <LOD | <LOD | <LOD | <LOD | <LOD |
| **F73** | <LOD | <LOD | <LOD | <LOD | 0,00016 | <LOD | <LOD | <LOD | <LOD | <LOD | <LOD |
| **F74** | <LOD | <LOD | <LOD | <LOD | 0,00323 | <LOD | <LOD | <LOD | <LOD | <LOD | <LOD |
| **F75** | <LOD | <LOD | <LOD | <LOD | 0,00021 | <LOD | <LOD | <LOD | <LOD | <LOD | <LOD |
| **F76** | <LOD | <LOD | <LOQ | <LOD | <LOD | <LOD | <LOD | <LOD | <LOD | <LOD | <LOD |
| **F77** | <LOD | <LOD | <LOD | <LOD | 0,00474 | <LOD | 0,00018 | <LOD | <LOQ | <LOD | <LOD |
| **F78** | <LOD | <LOD | <LOD | <LOD | 0,00009 | <LOD | <LOD | <LOD | <LOD | <LOD | <LOD |
| **F79** | <LOD | <LOD | <LOQ | <LOD | 0,00008 | <LOD | <LOD | <LOD | <LOD | <LOD | <LOD |
| **F80** | <LOD | <LOD | <LOQ | <LOD | 0,00008 | <LOD | <LOD | <LOD | <LOD | <LOD | <LOD |
| **F81** | <LOD | <LOD | <LOD | <LOD | 0,00032 | <LOD | <LOD | <LOD | <LOQ | <LOD | <LOD |
| **F82** | <LOD | <LOD | <LOQ | <LOD | 0,00011 | <LOD | <LOD | <LOD | <LOD | <LOD | <LOD |
| **F83** | <LOD | <LOD | <LOD | <LOD | 0,00358 | <LOD | 0,00037 | <LOQ | <LOD | <LOD | <LOD |
| **F84** | <LOD | <LOD | <LOD | <LOD | 0,00340 | <LOD | <LOD | <LOD | <LOD | <LOD | <LOD |
| **F85** | <LOD | <LOD | <LOQ | <LOD | 0,00008 | <LOD | <LOD | <LOD | <LOD | <LOD | <LOD |

| **Sample number** | **Hexaconazole** | **Metalaxyl** | **Piperonylbutoxide** | **Prochloraz** | **Propanil** | **Pyrimethanil** | **Tebuconazole** | **Tebufenozide** | **Triademifon** | **Triademinol** |
| --- | --- | --- | --- | --- | --- | --- | --- | --- | --- | --- |
| **EU allowed content [mg/L]** | 0,01 | 1 |  | 0,03 | 0,01 | 5 | 1 | 4 | 0,01 | 0,01 |
| **F01** | <LOD | <LOD | <LOD | 0,01634 | <LOD | <LOD | <LOD | <LOD | <LOD | <LOD |
| **F02** | <LOD | <LOD | <LOD | <LOD | <LOD | <LOD | <LOD | <LOD | <LOD | <LOD |
| **F03** | <LOD | 0,00239 | <LOQ | <LOD | <LOD | <LOD | <LOD | <LOD | <LOQ | <LOD |
| **F04** | <LOD | <LOD | <LOD | <LOD | <LOD | <LOD | <LOD | <LOD | <LOD | <LOD |
| **F05** | <LOD | <LOD | <LOD | <LOD | <LOQ | <LOD | <LOD | <LOD | <LOD | <LOD |
| **F06** | <LOD | <LOD | <LOD | <LOD | <LOQ | <LOD | <LOD | <LOD | <LOD | <LOD |
| **F07** | <LOD | <LOD | <LOQ | <LOD | <LOD | <LOD | <LOD | <LOD | <LOD | <LOD |
| **F08** | <LOD | <LOD | <LOQ | <LOD | <LOD | <LOD | <LOD | <LOD | <LOD | <LOD |
| **F09** | <LOD | 0,00051 | 0,00004 | <LOD | <LOD | <LOD | <LOD | <LOD | <LOQ | <LOD |
| **F10** | <LOQ | 0,00010 | <LOQ | <LOD | <LOQ | <LOD | <LOQ | <LOD | <LOD | <LOD |
| **F11** | <LOD | <LOD | 0,00008 | <LOD | <LOD | <LOD | <LOD | <LOD | <LOD | <LOD |
| **F12** | <LOD | <LOD | <LOD | <LOD | 0,00013 | <LOD | <LOD | <LOD | <LOD | <LOD |
| **F13** | <LOD | <LOQ | 0,00005 | <LOD | <LOD | <LOD | <LOD | <LOD | <LOD | <LOD |
| **F14** | <LOD | <LOD | <LOQ | <LOD | <LOD | <LOQ | <LOQ | <LOD | <LOD | <LOD |
| **F15** | <LOD | 0,00657 | <LOD | <LOD | <LOQ | <LOD | <LOD | <LOD | <LOD | <LOD |
| **F16** | <LOD | 0,01289 | <LOD | <LOD | <LOD | <LOD | <LOD | <LOD | <LOD | <LOD |
| **F17** | <LOD | <LOQ | <LOD | <LOD | <LOD | 0,00070 | 0,00152 | <LOQ | <LOD | 0,00120 |
| **F18** | <LOD | 0,00014 | <LOQ | <LOD | <LOD | <LOD | <LOD | <LOD | <LOD | <LOD |
| **F19** | <LOD | <LOD | <LOD | <LOD | <LOD | <LOD | <LOD | <LOD | <LOD | <LOD |
| **F20** | <LOD | 0,00011 | 0,00004 | <LOD | <LOD | <LOQ | <LOD | <LOD | <LOD | <LOD |
| **F21** | <LOD | 0,00043 | <LOD | <LOD | 0,00014 | <LOD | <LOD | <LOD | <LOD | 0,00063 |
| **F22** | <LOD | <LOQ | 0,00005 | <LOD | <LOD | <LOQ | <LOQ | 0,06853 | <LOD | <LOD |
| **F23** | <LOD | <LOD | <LOQ | <LOD | <LOD | <LOD | <LOD | 0,00026 | <LOD | <LOD |
| **F24** | 0,00084 | 0,00010 | <LOD | <LOD | <LOD | <LOD | <LOD | <LOQ | <LOD | <LOD |
| **F25** | <LOD | <LOQ | <LOQ | <LOD | <LOD | <LOD | <LOD | 0,00025 | <LOD | <LOD |
| **F26** | <LOD | <LOD | <LOD | <LOD | <LOD | <LOD | <LOD | <LOD | <LOD | <LOD |
| **F27** | <LOD | <LOD | <LOQ | <LOD | <LOD | <LOD | <LOD | <LOD | <LOD | <LOD |
| **F28** | <LOD | <LOD | <LOD | <LOD | <LOD | <LOD | <LOD | <LOD | <LOD | <LOD |
| **F29** | <LOD | <LOD | <LOQ | <LOD | <LOD | <LOD | <LOD | <LOD | <LOD | <LOD |
| **F30** | <LOD | 0,00049 | <LOD | <LOD | <LOD | <LOD | <LOD | <LOD | <LOD | <LOD |
| **F31** | <LOD | <LOD | <LOD | <LOD | <LOD | <LOD | <LOQ | <LOD | <LOD | <LOD |
| **F32** | <LOD | 0,00010 | 0,00004 | <LOD | <LOD | <LOD | <LOD | <LOD | <LOD | <LOD |
| **F33** | <LOD | 0,00103 | <LOD | <LOD | <LOD | <LOD | <LOD | <LOD | <LOD | 0,00004 |
| **F34** | <LOD | <LOD | <LOQ | <LOD | <LOD | <LOD | <LOD | <LOD | <LOD | <LOD |
| **F35** | <LOD | 0,00007 | <LOQ | <LOD | <LOD | 0,00546 | <LOQ | <LOD | <LOD | <LOD |
| **F36** | <LOQ | 0,00034 | <LOQ | <LOD | <LOD | 0,00015 | 0,00012 | 0,00026 | <LOD | <LOD |
| **F37** | <LOD | <LOD | <LOD | <LOD | <LOD | 0,00183 | 0,00250 | <LOD | <LOD | <LOD |
| **F38** | <LOD | <LOD | <LOQ | <LOD | <LOD | <LOD | <LOD | <LOD | <LOD | <LOD |
| **F39** | <LOD | 0,00061 | <LOD | <LOD | <LOD | <LOD | <LOD | <LOD | <LOQ | 0,00010 |
| **F40** | <LOD | 0,00014 | <LOD | <LOD | <LOD | <LOD | <LOD | <LOD | <LOQ | 0,00014 |
| **F41** | <LOD | 0,00007 | 0,00004 | <LOD | <LOD | <LOD | <LOD | <LOD | <LOD | 0,00006 |
| **F42** | <LOD | <LOD | <LOD | <LOD | <LOD | <LOD | <LOD | <LOD | <LOD | <LOD |
| **F43** | <LOD | <LOD | 0,00007 | <LOD | <LOD | 0,00063 | <LOQ | 0,00005 | <LOD | <LOD |
| **F44** | <LOD | 0,00010 | <LOD | <LOD | <LOD | <LOD | <LOD | <LOD | <LOD | 0,00004 |
| **F45** | <LOD | 0,00026 | <LOQ | <LOD | <LOD | 0,00030 | 0,00005 | 0,00021 | <LOD | 0,00010 |
| **F46** | 0,00026 | <LOQ | <LOQ | <LOD | <LOD | <LOQ | <LOQ | <LOQ | <LOD | <LOD |
| **F47** | <LOD | <LOD | 0,00005 | <LOD | <LOD | <LOD | 0,00038 | <LOD | 0,00012 | 0,00193 |
| **F48** | <LOD | <LOD | <LOD | <LOD | <LOD | 0,00013 | <LOD | <LOD | <LOD | <LOD |
| **F49** | <LOD | <LOD | <LOQ | <LOD | <LOD | <LOD | <LOD | <LOD | <LOD | <LOD |
| **F50** | 0,00009 | 0,00137 | <LOQ | <LOD | <LOD | <LOD | <LOD | <LOD | 0,00013 | 0,00051 |
| **F51** | <LOD | <LOD | <LOD | <LOD | <LOD | <LOD | <LOD | <LOD | <LOD | <LOD |
| **F52** | <LOD | <LOD | <LOD | <LOD | <LOD | 0,00107 | <LOD | <LOQ | <LOD | <LOD |
| **F53** | <LOD | <LOD | <LOD | <LOD | <LOD | <LOD | <LOD | <LOD | <LOD | <LOD |
| **F54** | <LOD | <LOD | <LOD | <LOD | <LOD | <LOD | <LOD | <LOD | 0,00009 | <LOD |
| **F56** | 0,00015 | <LOD | <LOQ | <LOD | <LOD | 0,00074 | 0,00008 | <LOD | <LOD | <LOD |
| **F57** | 0,00024 | <LOD | <LOD | <LOD | <LOD | 0,00448 | <LOD | <LOD | <LOD | <LOD |
| **F58** | <LOQ | <LOD | <LOD | <LOD | <LOD | 0,02191 | 0,00214 | <LOD | <LOD | <LOD |
| **F59** | <LOD | <LOD | <LOD | <LOD | <LOD | 0,01876 | <LOD | <LOQ | <LOD | <LOD |
| **F60** | <LOD | <LOD | <LOQ | <LOD | <LOD | 0,17278 | <LOD | <LOD | 0,00027 | <LOD |
| **F61** | <LOD | <LOD | <LOQ | <LOD | <LOD | 0,00037 | <LOD | 0,00012 | <LOD | <LOD |
| **F62** | <LOD | <LOD | <LOD | <LOD | <LOD | <LOD | <LOD | <LOD | <LOD | <LOD |
| **F63** | <LOD | <LOD | <LOD | <LOD | <LOD | <LOD | <LOD | <LOD | <LOD | <LOD |
| **F64** | <LOD | <LOD | <LOD | <LOD | <LOD | <LOD | <LOD | <LOD | <LOD | <LOD |
| **F65** | <LOD | <LOD | <LOD | <LOD | <LOD | <LOD | <LOD | <LOD | <LOD | <LOD |
| **F66** | <LOD | <LOD | <LOD | <LOD | <LOD | <LOQ | <LOD | <LOD | <LOD | <LOD |
| **F67** | <LOD | <LOQ | <LOD | <LOD | <LOD | 0,00068 | <LOD | <LOD | <LOD | <LOD |
| **F68** | <LOD | <LOD | <LOD | <LOD | <LOD | <LOD | <LOD | <LOD | <LOD | <LOD |
| **F69** | <LOD | <LOD | <LOD | <LOD | <LOD | <LOD | <LOD | <LOD | <LOD | <LOD |
| **F70** | <LOD | <LOD | <LOD | <LOD | <LOD | 0,00823 | 0,00008 | <LOD | <LOD | <LOD |
| **F71** | <LOD | <LOQ | <LOD | <LOD | <LOD | <LOD | <LOD | <LOD | <LOD | <LOD |
| **F72** | <LOD | <LOD | <LOD | <LOD | <LOD | <LOD | <LOD | <LOD | <LOD | <LOD |
| **F73** | <LOD | <LOD | <LOD | <LOD | <LOD | <LOD | <LOD | <LOD | <LOD | <LOD |
| **F74** | <LOD | 0,00174 | <LOD | <LOD | <LOD | <LOD | <LOD | <LOD | 0,00008 | <LOD |
| **F75** | <LOD | <LOD | <LOD | <LOD | <LOD | <LOD | <LOD | <LOD | <LOD | <LOD |
| **F76** | <LOD | <LOD | <LOD | <LOD | <LOD | <LOD | <LOD | <LOD | <LOD | <LOD |
| **F77** | <LOD | <LOQ | <LOD | <LOD | <LOD | <LOD | <LOD | <LOD | <LOD | <LOD |
| **F78** | <LOD | 0,00009 | <LOQ | <LOD | <LOD | <LOD | <LOD | <LOD | <LOD | <LOD |
| **F79** | <LOD | <LOD | <LOQ | <LOD | <LOD | <LOD | <LOD | <LOD | <LOD | <LOD |
| **F80** | <LOD | <LOD | <LOD | <LOD | <LOD | <LOD | <LOD | <LOD | <LOD | <LOD |
| **F81** | <LOD | <LOQ | <LOQ | <LOD | <LOD | 0,00034 | <LOD | <LOD | <LOD | <LOD |
| **F82** | <LOD | 0,00009 | <LOD | <LOD | <LOD | <LOD | <LOD | <LOD | <LOD | <LOD |
| **F83** | <LOD | <LOD | <LOQ | <LOD | <LOD | <LOD | <LOD | <LOD | <LOD | <LOD |
| **F84** | <LOD | 0,00007 | <LOQ | <LOD | <LOD | <LOD | <LOD | <LOD | <LOD | <LOD |
| **F85** | 0,00009 | 0,00019 | <LOQ | <LOD | <LOD | <LOD | <LOD | <LOD | <LOD | <LOD |
