# Supplementary material for: The temporal variation in pesticide concentrations within matured French wines
Source: PLoS One. 2025 Feb 11;20(2):e0317086. doi: 10.1371/journal.pone.0317086 (PMC11813125; doi:10.1371/journal.pone.0317086)
Supplement: S7 Table — (DOCX) [file pone.0317086.s007.docx]

**Table S7 The residues (mg/L) of pesticides detected in the wine sediment of wine samples (in green values above 0.01mg/L, in yellow values above 0,05mg/L)**

| **Sample number** | **Atrazine** | **Azoxy**  **strobine** | **Benalaxyl** | **Cadusa**  **fos** | **Carbaryl** | **Carben**  **dazim** | **Dietho**  **fenocarb** | **Difeno**  **conazole** | **Dimethomorph** | **Meta**  **laxyl** | **Pyrime**  **thanil** | **Tebuco**  **nazole** | **Tebufenozide** |
| --- | --- | --- | --- | --- | --- | --- | --- | --- | --- | --- | --- | --- | --- |
| **EU allowed content [mg/L]** | x | 3.0 | x | 0,01 | 0,01 | 0,5 | 0,01 | 3.0 | 3.0 | 1.0 | 5.0 | 1 | 4.0 |
| **F01** | <LOD | <LOD | <LOD | <LOD | 0,89 | <LOD | <LOD | <LOQ | <LOD | <LOD | <LOD | <LOD | <LOD |
| **F02** | <LOD | <LOD | <LOD | 0,05 | <LOD | 0,09 | <LOD | <LOD | <LOD | <LOD | <LOD | <LOD | <LOD |
| **F03** | <LOD | <LOD | <LOD | 0,02 | <LOD | 1,11 | <LOD | <LOD | <LOD | 0,02 | <LOD | <LOD | <LOD |
| **F04** | <LOD | <LOD | <LOD | 0,01 | <LOD | <LOD | <LOD | <LOD | <LOD | <LOD | <LOD | <LOD | <LOD |
| **F05** | <LOD | <LOD | <LOD | <LOQ | 0,15 | 0,10 | <LOD | <LOD | <LOD | <LOD | <LOD | <LOD | <LOD |
| **F06** | <LOD | <LOD | <LOD | 0,06 | <LOD | 0,46 | <LOD | <LOD | <LOD | <LOD | <LOD | <LOD | <LOD |
| **F07** | <LOD | <LOD | <LOD | <LOQ | <LOD | 0,17 | <LOD | <LOD | <LOD | <LOD | <LOD | <LOD | <LOD |
| **F08** | <LOD | <LOD | <LOD | 0,04 | <LOD | <LOD | <LOD | <LOD | <LOD | <LOD | <LOD | <LOD | <LOD |
| **F09** | <LOD | <LOD | <LOD | 0,06 | <LOD | <LOQ | <LOD | <LOD | <LOD | <LOD | <LOD | <LOD | <LOD |
| **F10** | <LOD | <LOD | <LOD | 0,03 | <LOD | <LOQ | <LOD | <LOD | <LOD | <LOD | <LOD | <LOD | <LOD |
| **F11** | <LOD | <LOD | <LOD | <LOQ | <LOD | <LOD | <LOD | <LOD | <LOD | <LOD | <LOD | <LOD | <LOD |
| **F12** | <LOD | <LOD | <LOD | 0,05 | <LOD | <LOD | <LOD | <LOD | <LOD | <LOD | <LOD | <LOD | <LOD |
| **F13** | <LOD | <LOD | <LOD | <LOQ | <LOD | 1,23 | <LOQ | <LOD | <LOQ | <LOD | <LOD | <LOD | <LOD |
| **F14** | <LOD | <LOD | <LOD | <LOQ | <LOD | <LOQ | <LOD | <LOD | <LOD | 0,01 | <LOD | <LOD | <LOD |
| **F15** | <LOD | <LOD | <LOQ | 0,07 | <LOD | 0,04 | <LOD | <LOD | <LOQ | <LOD | <LOQ | <LOD | <LOD |
| **F16** | <LOD | <LOD | <LOD | <LOQ | <LOD | <LOQ | <LOD | <LOD | <LOD | 0,03 | <LOD | <LOD | <LOD |
| **F17** | <LOD | <LOD | <LOD | <LOD | <LOD | <LOD | <LOD | <LOD | <LOD | <LOD | <LOD | <LOQ | <LOD |
| **F18** | <LOD | <LOD | <LOD | <LOQ | <LOD | <LOD | <LOD | <LOD | <LOD | <LOD | <LOD | <LOD | <LOD |
| **F19** | <LOD | <LOD | <LOD | <LOQ | <LOD | <LOD | <LOD | <LOD | <LOD | <LOD | <LOD | <LOD | <LOD |
| **F20** | <LOD | <LOD | <LOD | <LOD | <LOD | <LOD | <LOD | <LOD | <LOD | <LOD | <LOD | <LOD | <LOD |
| **F21** | <LOD | <LOD | <LOD | <LOQ | <LOD | 0,05 | <LOD | <LOD | <LOD | <LOQ | <LOD | <LOD | <LOD |
| **F22** | <LOD | <LOD | <LOD | <LOD | <LOD | <LOD | <LOD | <LOQ | 0,31 | <LOD | <LOD | <LOD | 0,24 |
| **F23** | <LOD | <LOD | <LOD | <LOD | <LOD | 0,06 | <LOD | 0,01 | <LOD | <LOD | <LOD | <LOD | <LOD |
| **F24** | <LOD | <LOD | <LOD | <LOD | <LOD | 0,63 | <LOD | <LOD | <LOD | <LOD | <LOD | <LOD | <LOD |
| **F25** | <LOD | <LOD | <LOD | <LOD | <LOD | 0,30 | <LOD | <LOD | <LOD | <LOD | <LOD | <LOD | <LOD |
| **F26** | <LOD | <LOD | <LOD | <LOD | <LOD | <LOD | <LOD | <LOD | <LOD | <LOD | <LOD | 0,08 | <LOD |
| **F27** | <LOD | <LOD | <LOD | <LOQ | <LOD | <LOD | <LOD | <LOD | <LOD | <LOD | <LOD | <LOD | <LOD |
| **F28** | <LOD | <LOD | <LOD | <LOQ | <LOD | <LOD | <LOD | <LOD | <LOD | <LOD | <LOD | <LOD | <LOD |
| **F29** | <LOD | <LOD | <LOD | <LOQ | 9,37 | <LOD | <LOD | <LOD | <LOD | <LOD | <LOD | <LOD | <LOD |
| **F30** | <LOD | <LOD | <LOD | <LOD | <LOD | 0,09 | <LOD | <LOD | <LOD | <LOQ | <LOD | <LOD | <LOD |
| **F31** | <LOD | <LOD | <LOD | <LOD | 0,01 | 0,01 | <LOD | 0,02 | <LOD | <LOD | <LOD | <LOD | <LOD |
| **F32** | <LOD | <LOD | <LOD | <LOD | <LOD | 0,08 | <LOD | <LOQ | <LOD | <LOD | <LOD | <LOD | <LOD |
| **F33** | <LOD | <LOD | <LOD | <LOD | 0,04 | 0,10 | <LOD | <LOD | <LOD | <LOQ | <LOD | <LOD | <LOD |
| **F34** | <LOD | <LOD | <LOD | <LOD | <LOD | <LOD | <LOD | <LOD | <LOD | <LOD | <LOD | <LOD | <LOD |
| **F35** | <LOD | <LOD | <LOD | <LOD | <LOD | <LOQ | <LOD | <LOD | <LOD | <LOD | 0,12 | <LOD | <LOD |
| **F36** | <LOD | <LOD | <LOD | <LOD | <LOD | 0,20 | <LOD | <LOD | 0,06 | <LOD | <LOD | <LOD | <LOD |
| **F37** | <LOD | <LOD | <LOD | <LOD | <LOD | <LOD | <LOD | <LOD | <LOD | <LOD | 0,02 | 0,02 | <LOD |
| **F38** | <LOD | <LOD | <LOD | <LOQ | <LOD | 0,05 | <LOD | <LOD | <LOD | <LOD | <LOD | <LOD | <LOD |
| **F39** | <LOD | <LOD | <LOD | <LOD | <LOD | <LOQ | <LOD | <LOD | <LOD | <LOD | <LOD | <LOD | <LOD |
| **F40** | <LOD | <LOD | <LOD | <LOD | <LOD | <LOD | <LOD | <LOD | <LOD | <LOD | <LOD | <LOD | <LOD |
| **F41** | <LOD | <LOD | <LOD | <LOD | <LOD | <LOD | <LOD | <LOD | <LOD | <LOD | <LOD | <LOD | <LOD |
| **F42** | <LOD | <LOD | <LOD | <LOD | <LOQ | <LOD | <LOD | <LOD | <LOD | <LOD | <LOD | <LOD | <LOD |
| **F43** | <LOD | <LOQ | <LOD | <LOD | <LOD | <LOD | <LOD | <LOD | <LOD | <LOD | <LOQ | <LOD | <LOD |
| **F44** | <LOD | <LOD | <LOD | <LOD | <LOD | <LOQ | <LOD | <LOD | <LOD | <LOD | <LOD | <LOD | <LOD |
| **F45** | <LOD | <LOD | <LOD | <LOD | <LOD | 0,07 | <LOD | <LOD | <LOD | <LOD | <LOD | <LOD | <LOD |
| **F46** | <LOQ | <LOD | <LOD | <LOD | <LOD | 0,19 | <LOD | <LOD | <LOD | <LOD | <LOD | <LOD | <LOD |
| **F47** | <LOD | <LOD | <LOD | <LOD | <LOQ | 0,02 | <LOD | <LOD | 0,23 | <LOD | <LOD | 0,01 | <LOD |
| **F48** | <LOD | <LOD | <LOD | <LOD | <LOD | 0,03 | <LOD | <LOD | <LOD | <LOD | <LOQ | <LOD | <LOD |
| **F49** | <LOD | <LOD | <LOD | <LOQ | 0,04 | 0,14 | <LOD | <LOD | <LOD | <LOD | <LOD | <LOD | <LOD |
| **F50** | <LOD | <LOD | <LOD | <LOD | <LOD | 0,04 | <LOD | <LOD | <LOD | <LOQ | <LOD | <LOD | <LOD |
| **F51** | <LOD | <LOD | <LOD | <LOD | <LOD | 0,41 | <LOD | <LOQ | <LOQ | <LOD | <LOD | <LOD | <LOD |
| **F52** | <LOD | <LOD | <LOD | <LOD | <LOD | 2,88 | 0,01 | <LOD | <LOQ | <LOQ | 0,03 | <LOD | <LOD |
| **F53** | <LOD | <LOD | <LOD | <LOD | <LOD | 1,40 | <LOD | <LOD | <LOD | <LOD | <LOD | <LOD | <LOD |
| **F54** | <LOD | <LOD | <LOD | <LOQ | <LOD | <LOD | <LOD | <LOD | <LOD | <LOQ | <LOD | <LOD | <LOD |
| **F56** | <LOD | <LOD | <LOD | <LOD | <LOD | 0,12 | <LOD | <LOD | 0,05 | <LOD | <LOQ | <LOD | <LOD |
| **F57** | <LOD | <LOD | <LOD | <LOD | <LOD | 0,07 | <LOD | <LOD | <LOD | <LOD | 0,09 | <LOD | <LOD |
| **F58** | <LOD | <LOD | <LOD | <LOD | <LOD | <LOQ | <LOD | <LOD | <LOQ | <LOD | 0,51 | 0,03 | <LOD |
| **F59** | <LOD | <LOD | <LOD | <LOD | <LOD | 1,07 | <LOQ | <LOD | <LOD | <LOD | 0,58 | <LOD | <LOD |
| **F60** | <LOD | 0,12 | <LOD | <LOD | <LOD | 1,83 | 0,02 | <LOD | <LOD | <LOD | 1,74 | <LOD | <LOD |
| **F61** | <LOD | <LOD | <LOD | <LOD | <LOD | 0,90 | <LOQ | <LOD | <LOD | <LOD | <LOQ | <LOD | <LOD |
| **F62** | <LOD | <LOD | <LOD | <LOQ | <LOD | <LOD | <LOD | <LOD | <LOD | <LOD | <LOD | <LOD | <LOD |
| **F63** | <LOD | <LOD | <LOD | <LOQ | <LOD | 4,51 | <LOD | <LOD | <LOD | <LOD | <LOD | <LOD | <LOD |
| **F64** | <LOD | <LOD | <LOD | <LOD | <LOD | <LOD | <LOD | <LOD | <LOD | <LOD | <LOD | <LOD | <LOD |
| **F65** | <LOD | <LOD | <LOD | <LOD | <LOD | <LOD | <LOD | <LOD | <LOD | <LOD | <LOD | <LOD | <LOD |
| **F66** | <LOD | <LOD | <LOD | <LOD | <LOD | 0,06 | <LOD | <LOD | <LOD | <LOD | <LOD | <LOD | <LOD |
| **F67** | <LOD | <LOD | <LOD | <LOD | <LOD | <LOD | <LOD | <LOD | <LOD | <LOD | <LOD | <LOD | <LOD |
| **F68** | <LOD | <LOD | <LOD | <LOD | <LOD | <LOD | <LOD | <LOD | <LOD | <LOD | <LOD | <LOD | <LOD |
| **F69** | <LOD | <LOD | <LOD | <LOQ | <LOD | 0,79 | <LOD | <LOD | <LOD | <LOD | <LOD | <LOD | <LOD |
| **F70** | <LOD | <LOD | <LOD | <LOD | <LOD | 0,14 | <LOD | <LOD | <LOD | <LOD | 0,13 | <LOD | <LOD |
| **F71** | <LOD | <LOD | <LOD | <LOQ | <LOD | 0,02 | <LOD | <LOD | <LOD | <LOD | <LOD | <LOD | <LOD |
| **F72** | <LOD | <LOD | <LOD | <LOD | <LOD | <LOD | <LOD | <LOD | <LOD | <LOD | <LOD | <LOD | <LOD |
| **F73** | <LOD | <LOD | <LOD | <LOD | <LOD | <LOD | <LOD | <LOD | <LOD | <LOD | <LOD | <LOD | <LOD |
| **F74** | <LOD | <LOD | <LOD | <LOD | <LOD | 0,12 | <LOD | <LOD | <LOD | <LOQ | <LOD | <LOD | <LOD |
| **F75** | <LOD | <LOD | <LOD | <LOQ | <LOD | <LOD | <LOD | <LOD | <LOD | <LOD | <LOD | <LOD | <LOD |
| **F76** | <LOD | <LOD | <LOD | <LOD | <LOQ | <LOD | <LOD | <LOD | <LOD | <LOD | <LOD | <LOD | <LOD |
| **F77** | <LOD | <LOD | <LOD | <LOD | <LOQ | 0,20 | <LOD | <LOD | <LOD | <LOD | <LOD | <LOD | <LOD |
| **F78** | <LOD | <LOD | <LOD | <LOD | <LOD | <LOD | <LOD | <LOD | <LOD | <LOD | <LOD | <LOD | <LOD |
| **F79** | <LOD | <LOD | <LOD | <LOQ | <LOD | <LOD | <LOD | <LOD | <LOD | <LOD | <LOD | <LOD | <LOD |
| **F80** | <LOD | <LOD | <LOD | <LOD | <LOD | <LOD | <LOD | <LOD | <LOD | <LOD | <LOD | <LOD | <LOD |
| **F81** | <LOD | <LOD | <LOD | <LOD | <LOD | <LOQ | <LOD | <LOD | <LOD | <LOD | 0,01 | <LOD | <LOD |
| **F82** | <LOD | <LOD | <LOD | <LOD | <LOD | <LOD | <LOD | <LOD | <LOD | <LOD | <LOD | <LOD | <LOD |
| **F83** | <LOD | <LOD | <LOD | <LOD | <LOD | 0,26 | <LOD | <LOQ | <LOD | <LOD | <LOD | <LOD | <LOD |
| **F84** | <LOD | <LOD | <LOD | <LOD | <LOD | 0,35 | <LOD | <LOD | <LOD | <LOD | <LOD | <LOD | <LOD |
| **F85** | <LOD | <LOD | <LOD | <LOD | <LOD | <LOD | <LOD | <LOD | <LOD | <LOQ | <LOD | <LOD | <LOD |
