# Supplementary material for: The temporal variation in pesticide concentrations within matured French wines
Source: PLoS One. 2025 Feb 11;20(2):e0317086. doi: 10.1371/journal.pone.0317086 (PMC11813125; doi:10.1371/journal.pone.0317086)
Supplement: S9 Table — (DOCX) [file pone.0317086.s009.docx]

**Table S9 The amount of pesticides found per bottle of wine in the wine sediment**

| **Sample number** | **<LOQ and >LOD per bottle** | **>LOQ per bottle** | **sum per bottle** |
| --- | --- | --- | --- |
| **F01** | 1 | 1 | 2 |
| **F02** | 0 | 2 | 2 |
| **F03** | 0 | 3 | 3 |
| **F04** | 0 | 1 | 1 |
| **F05** | 1 | 2 | 3 |
| **F06** | 0 | 2 | 2 |
| **F07** | 1 | 1 | 2 |
| **F08** | 0 | 1 | 1 |
| **F09** | 1 | 1 | 2 |
| **F10** | 1 | 1 | 2 |
| **F11** | 1 | 0 | 1 |
| **F12** | 0 | 1 | 1 |
| **F13** | 3 | 1 | 4 |
| **F14** | 2 | 1 | 3 |
| **F15** | 3 | 2 | 5 |
| **F16** | 2 | 1 | 3 |
| **F17** | 1 | 0 | 1 |
| **F18** | 1 | 0 | 1 |
| **F19** | 1 | 0 | 1 |
| **F20** | 0 | 0 | 0 |
| **F21** | 2 | 1 | 3 |
| **F22** | 1 | 2 | 3 |
| **F23** | 0 | 2 | 2 |
| **F24** | 0 | 1 | 1 |
| **F25** | 0 | 1 | 1 |
| **F26** | 0 | 1 | 1 |
| **F27** | 1 | 0 | 1 |
| **F28** | 1 | 0 | 1 |
| **F29** | 1 | 1 | 2 |
| **F30** | 1 | 1 | 2 |
| **F31** | 0 | 3 | 3 |
| **F32** | 1 | 1 | 2 |
| **F33** | 1 | 2 | 3 |
| **F34** | 0 | 0 | 0 |
| **F35** | 1 | 1 | 2 |
| **F36** | 0 | 2 | 2 |
| **F37** | 0 | 2 | 2 |
| **F38** | 1 | 1 | 2 |
| **F39** | 1 | 0 | 1 |
| **F40** | 0 | 0 | 0 |
| **F41** | 0 | 0 | 0 |
| **F42** | 1 | 0 | 1 |
| **F43** | 2 | 0 | 2 |
| **F44** | 1 | 0 | 1 |
| **F45** | 0 | 1 | 1 |
| **F46** | 1 | 1 | 2 |
| **F47** | 1 | 3 | 4 |
| **F48** | 1 | 1 | 2 |
| **F49** | 1 | 2 | 3 |
| **F50** | 1 | 1 | 2 |
| **F51** | 2 | 1 | 3 |
| **F52** | 2 | 3 | 5 |
| **F53** | 0 | 1 | 1 |
| **F54** | 2 | 0 | 2 |
| **F56** | 1 | 2 | 3 |
| **F57** | 0 | 2 | 2 |
| **F58** | 2 | 2 | 4 |
| **F59** | 1 | 2 | 3 |
| **F60** | 0 | 4 | 4 |
| **F61** | 2 | 1 | 3 |
| **F62** | 1 | 0 | 1 |
| **F63** | 1 | 1 | 2 |
| **F64** | 0 | 0 | 0 |
| **F65** | 0 | 0 | 0 |
| **F66** | 0 | 1 | 1 |
| **F67** | 0 | 0 | 0 |
| **F68** | 0 | 0 | 0 |
| **F69** | 1 | 1 | 2 |
| **F70** | 0 | 2 | 2 |
| **F71** | 1 | 1 | 2 |
| **F72** | 0 | 0 | 0 |
| **F73** | 0 | 0 | 0 |
| **F74** | 1 | 1 | 2 |
| **F75** | 1 | 0 | 1 |
| **F76** | 1 | 0 | 1 |
| **F77** | 1 | 1 | 2 |
| **F78** | 0 | 0 | 0 |
| **F79** | 1 | 0 | 1 |
| **F80** | 0 | 0 | 0 |
| **F81** | 1 | 1 | 2 |
| **F82** | 0 | 0 | 0 |
| **F83** | 1 | 1 | 2 |
| **F84** | 0 | 1 | 1 |
| **F85** | 1 | 0 | 1 |
|  |  |  |  |
| **Average** | 0.76 | 0.96 | 1.73 |
