# Supplementary material for: The temporal variation in pesticide concentrations within matured French wines
Source: PLoS One. 2025 Feb 11;20(2):e0317086. doi: 10.1371/journal.pone.0317086 (PMC11813125; doi:10.1371/journal.pone.0317086)
Supplement: S10 Table — (DOCX) [file pone.0317086.s010.docx]

**Table S10 The copper concentrations detected, per wine sample (mg/L)**

| **Sample number** | **Copper (mg/L)** | **Sample number** | **Copper (mg/L)** |
| --- | --- | --- | --- |
| F2 | 0.428 | F42 | <0.025 |
| F3 | 0.044 | F43 | <0.025 |
| F4 | 0.430 | F44 | 0.105 |
| F5 | 0.177 | F45 | <0.025 |
| F6 | <0.025 | F46 | 0.136 |
| F7 | 0.038 | F47 | <0.025 |
| F8 | 0.031 | F48 | 0.090 |
| F9 | 0.028 | F49 | 0.047 |
| F10 | 0.044 | F50 | 0.150 |
| F11 | <0.025 | F51 | 0.138 |
| F12 | <0.025 | F52 | 0.082 |
| F13 | <0.025 | F53 | 0.150 |
| F14 | 0.068 | F54 | 0.098 |
| F15 | 0.127 | F56 | 0.278 |
| F16 | 0.644 | F57 | 0.234 |
| F17 | 0.076 | F58 | 0.268 |
| F18 | 0.066 | F59 | 0.021 |
| F19 | <0.025 | F60 | 0.020 |
| F20 | <0.025 | F62 | 0.581 |
| F21 | 0.035 | F63 | 0.104 |
| F22 | 0.033 | F64 | 0.513 |
| F23 | 0.221 | F65 | 0.171 |
| F24 | 0.055 | F66 | 0.513 |
| F25 | <0.025 | F67 | 0.348 |
| F26 | 0.156 | F68 | 0.034 |
| F27 | <0.025 | F69 | 0.291 |
| F28 | 0.150 | F70 | 0.285 |
| F29 | 0.415 | F71 | 0.180 |
| F30 | 0.942 | F72 | 0.855 |
| F31 | 0.285 | F73 | 0.307 |
| F32 | 0.494 | F74 | 0.032 |
| F33 | 0.192 | F75 | 0.288 |
| F34 | 0.996 | F76 | 0.702 |
| F35 | <0.025 | F77 | 0.203 |
| F36 | <0.025 | F80 | 0.714 |
| F37 | <0.025 | F81 | 2.080 |
| F38 | <0.025 | F82 | 0.636 |
| F39 | 0.121 | F83 | 1.308 |
| F40 | 0.077 | F84 | 1.154 |
| F41 | <0.025 | F85 | 0.306 |
