# Supplementary material for: The temporal variation in pesticide concentrations within matured French wines
Source: PLoS One. 2025 Feb 11;20(2):e0317086. doi: 10.1371/journal.pone.0317086 (PMC11813125; doi:10.1371/journal.pone.0317086)
Supplement: S11 Table — (DOCX) [file pone.0317086.s011.docx]

**Table S11 The maximum residue values found in one analyzed bottle compared to ADI values and ARfD values for each pesticide (average weight female: 66.7kg. average weight male: 99kg)**

| **Pesticide** | **Maximum residue value detected in one of the analyzed wine bottles (µg/L wine** **)** | **Average consumption of pesticide [mg]** | **Frequent consumption of pesticide [mg]** |
| --- | --- | --- | --- |
| **Azoxystrobin** | 11.365 | 0.003 | 0.007 |
| **Benalaxyl** | 0.556 | 0.000 | 0.000 |
| **Carbaryl** | 2950.718 | 0.885 | 1.927 |
| **Carbendazim** | 34.799 | 0.010 | 0.023 |
| **Chlorpropham** | 0.474 | 0.000 | 0.000 |
| **Diethofencarb** | 7.942 | 0.002 | 0.005 |
| **Difenoconazole** | 2.469 | 0.001 | 0.002 |
| **Dimethomorph** | 12.296 | 0.004 | 0.008 |
| **Diuron** | 0.289 | 0.000 | 0.000 |
| **Fenbuconazole** | 0.091 | 0.000 | 0.000 |
| **Hexaconazole** | 0.844 | 0.000 | 0.001 |
| **Metalaxyl** | 12.890 | 0.004 | 0.008 |
| **Piperonylbutoxide** | 0.083 | 0.000 | 0.000 |
| **Prochloraz** | 16.345 | 0.005 | 0.011 |
| **Propanil** | 0.143 | 0.000 | 0.000 |
| **Pyrimethanil** | 172.778 | 0.052 | 0.113 |
| **Tebuconazole** | 2.498 | 0.001 | 0.002 |
| **Tebufenozide** | 68.533 | 0.021 | 0.045 |
| **Triademifon** | 0.272 | 0.000 | 0.000 |
| **Triademinol** | 1.934 | 0.001 | 0.001 |

| **Pesticide** | **ADI (mg/(kgBW*day))** | **% ADI for female average consumption** | **% ADI for female frequent consumption** | **% ADI for male average consumption** | **% ADI for male frequent consumption** | **ARfD (mg/kg BW/day)** | **% ARfD for female average consumption** | **% ARfD for female frequent consumption** | **% ARfD for male average consumption** | **% ARfD for male frequent consumption** |
| --- | --- | --- | --- | --- | --- | --- | --- | --- | --- | --- |
| **Azoxystrobin** | 0.20 | 0.03% | 0.06% | 0.02% | 0.05% | - | - | - | - | - |
| **Benalaxyl** | 0.04 | 0.01% | 0.01% | 0.01% | 0.01% | - | - | - | - | - |
| **Carbaryl** | 0.01 | 176.95% | 385.17% | 149.40% | 325.20% | 0.01 | 132.72% | 288.88% | 112.05% | 243.90% |
| **Carbendazim** | 0.02 | 0.78% | 1.70% | 0.66% | 1.44% | 0.02 | 0.78% | 1.70% | 0.66% | 1.44% |
| **Chlorpropham** | 0.05 | 0.00% | 0.01% | 0.00% | 0.01% | 0.50 | 0.00% | 0.00% | 0.00% | 0.00% |
| **Diethofencarb** | 0.43 | 0.01% | 0.02% | 0.01% | 0.02% | 0.03 | 0.12% | 0.26% | 0.10% | 0.22% |
| **Difenoconazole** | 0.01 | 0.11% | 0.24% | 0.09% | 0.20% | 0.20 | 0.01% | 0.01% | 0.00% | 0.01% |
| **Dimethomorph** | 0.05 | 0.11% | 0.24% | 0.09% | 0.20% | 0.60 | 0.01% | 0.02% | 0.01% | 0.02% |
| **Diuron** | 0.01 | 0.02% | 0.04% | 0.02% | 0.03% | 0.02 | 0.01% | 0.02% | 0.01% | 0.01% |
| **Fenbuconazole** | 0.01 | 0.01% | 0.01% | 0.01% | 0.01% | 0.30 | 0.00% | 0.00% | 0.00% | 0.00% |
| **Hexaconazole** | 0.01 | 0.08% | 0.17% | 0.06% | 0.14% | 0.03 | 0.02% | 0.03% | 0.01% | 0.03% |
| **Metalaxyl** | 0.08 | 0.07% | 0.16% | 0.06% | 0.13% | 0.50 | 0.01% | 0.03% | 0.01% | 0.02% |
| **Piperonylbutoxide** | - | - | - | - | - | - | - | - | - | - |
| **Prochloraz** | 0.01 | 0.74% | 1.60% | 0.62% | 1.35% | 0.02 | 0.43% | 0.94% | 0.37% | 0.79% |
| **Propanil** | 0.02 | 0.00% | 0.01% | 0.00% | 0.01% | - | - | - | - | - |
| **Pyrimethanil** | 0.17 | 0.46% | 1.00% | 0.39% | 0.84% | - | - | - | - | - |
| **Tebuconazole** | 0.03 | 0.04% | 0.08% | 0.03% | 0.07% | 0.03 | 0.04% | 0.08% | 0.03% | 0.07% |
| **Tebufenozide** | 0.02 | 1.54% | 3.35% | 1.30% | 2.83% | 0.90 | 0.03% | 0.07% | 0.03% | 0.06% |
| **Triademifon** | 0.03 | 0.00% | 0.01% | 0.00% | 0.01% | - | - | - | - | - |
| **Triademinol** | 0.05 | 0.02% | 0.04% | 0.01% | 0.03% | - | - | - | - | - |
