# Supplementary material for: The temporal variation in pesticide concentrations within matured French wines
Source: PLoS One. 2025 Feb 11;20(2):e0317086. doi: 10.1371/journal.pone.0317086 (PMC11813125; doi:10.1371/journal.pone.0317086)
Supplement: S12 Table — (DOCX) [file pone.0317086.s012.docx]

**Table S12 List of pesticides used and validated in the analytical method, and monitored across all wine samples**

| **Acephate** | **Fenoxycarb** | **Propanil** |
| --- | --- | --- |
| **Acetamiprid** | **Fenpropimorf** | **Propazine** |
| **Amethryn** | **Fenpyroximat** | **Propiconazole** |
| **Atrazine** | **Flazasulfuron** | **Propoxur** |
| **Azoxystrobine** | **Fluazufop-p-butyl** | **Prosulfocarb** |
| **Benalaxyl** | **Fludioxonil** | **Prosulfocarb** |
| **Bentazon** | **Flufenacet** | **Pyrachlostrobine** |
| **Bitertanol** | **Hexaconazole** | **Pyrazosulfuron-ethyl** |
| **Boscalid** | **Hexythiazox** | **Pyridaben** |
| **Butachlor** | **Imazalil** | **Pyrimethanil** |
| **Cadusafos** | **Imidacloprid** | **Sethoxydim** |
| **Carbaryl** | **Indoxacarb** | **Simazine** |
| **Carbendazim** | **Iprodione** | **Spinosad a** |
| **Carbetamide** | **Isoproturon** | **Spinosad d** |
| **Carbofuran** | **Kresoxim-methyl** | **Spirodiclofen** |
| **Carbosulfan** | **Linuron** | **Spiroxamine** |
| **Carfentrazon-ethyl** | **Malathion** | **Tau fluvalinate** |
| **Chlorotoluron** | **Metalaxyl** | **Tebuconazole** |
| **Chlorprofam** | **Metamitron** | **Tebufenozide** |
| **Chlorpyrifos** | **Metazochlor** | **Tebuthiuron** |
| **Clopyralid** | **Methabenzthiazuron** | **Temephos** |
| **Cyanizine** | **Methiocarb** | **Terbutryn** |
| **Cyflufenamid** | **Methomyl** | **Terbutylazine** |
| **Cymoxanil** | **Methoxifenozide** | **Terufos** |
| **Cyprodinil** | **Methribuzin** | **Thiabendazole** |
| **Diazinon** | **Metsulfuron-methyl** | **Thiacloprid** |
| **Diethofencarb** | **Monocrotophos** | **Thiametoxam** |
| **Difenacoum** | **Nicosulfuron** | **Thifensulfuron-methyl** |
| **Difenconazole** | **Oxamyl** | **Thiodicarb** |
| **Diflubenzuron** | **Parathion** | **Thiofanate-methyl** |
| **Dimethoate** | **Penconazole** | **Tirazophos** |
| **Dimethomorph** | **Pendimehtanil** | **Triademinol** |
| **Diuron** | **Piperonylbutoxide** | **Triadimefon** |
| **Epoxiconazole** | **Pirimicarb** | **Trifloxystrobine** |
| **Ethoprophos** | **Pirimiphos-methyl** | **Triticonazole** |
| **Fenamiphos** | **Prochloraz** | **Zoxamide** |
| **Fenbuconazole** | **Profenofos** |  |
