# Supplementary material for: The temporal variation in pesticide concentrations within matured French wines
Source: PLoS One. 2025 Feb 11;20(2):e0317086. doi: 10.1371/journal.pone.0317086 (PMC11813125; doi:10.1371/journal.pone.0317086)
Supplement: Attachment — Submitted filename: PONE-D-24-17146.docx [file pone.0317086.s013.docx]

Introduction:

Some of the key points may be discussed in introduction;

- Status of MRLs of pesticide residues in wine and wine grapes. The importance of processing factor/transfer rate and their relation to wine MRLs
- A brief review on analytical methods followed for pesticide residue analysis in wine may be discussed
- The authors made a detailed risk assessment of positive detection, but introduction does not see any such insight.
- The number of pesticides included in the study may be provided in the objective.

2 Material and methods:

The materials and methods should address the following;

- The chemicals and reagents used in the study along with their source; specifically, the pesticide standards (CRMs) used in the study should be provided.
- Did the authors analyzed red wine and white wine separately, are they used same procedure for both wine, is there any difference with respect to LOD and LOQ?
- The recovery specified for many pesticides studied are less than 50%., Did the authors applied recovery correction factor in calculating the residues? Why this much low recovery was observed? Did the authors calculate matrix effect? Is this low recovery being due to matrix suppression?
- What is the spiking concentrations where the authors did the recovery study? Which are the concentrations spiked for recovery study? One recovery data should necessarily be at LOQ level.
- How the authors established the LOD and LOQ for each analytes? Why the matrix effect did not calculate? How the quantification was performed in LC-MS, Matrix match calibration, or matrix based calibration or solvent calibration? These points should be included.
- Approximately, what is weight of sediments observed in the 50 mL wine? How much dilution factor applied for extraction in 15 mL CAN. Should be provided.
- Table A.2 and Table A.3 may be provided with retention time and mass transitions (Quantifier and Qualifier). All these pesticides are only tested or detected? If detected, should give detailed data about the list of pesticides tested.

(Overall, there is a lack of required information on analytical method followed, need further detailed information on analytical protocols as detailed above).

3.Results and discussion:

3.1.1. PPPs detected in old wines:

3.1.2. Number of PPPs detected in analyzed wine samples:

- How the classification of sample has been performed to old wines? It’s confusing what is old wines, analyzed wine, Are these data overlapping??

- The authors are made a detailed classification of the results based on plant protection products. However, some of the meaningful insight is missing such as; are these pesticides detected where officially allowed to use at the time of wine preparation?

- If any residue presents in old wine, what is the persistence of that pesticides in wine? Can we correlate that with persistence of pesticides?

- Authors may have a detailed discussion on MRLs of pesticide in wine and its compliance in tested wine.

- As per the results, less number of pesticides are detected in sediments which is ideally not expected since, the residues of pesticides will be more adsorbed in solid particles especially to the leese. Hence, this result may please be explained based on solubility of the pesticides in water/wine and octanol/water coefficient (log Kow values).

- The study is only focused on Belgian population and pesticides registered in Belgium is only considered. Is this is aligned with EU regulation? As per mu understanding, the list of pesticides covered under this study does not cover most of the pesticides used in EU countries. Is this work has only local importance?

-3.3. Copper in old wines: I the limit regulated in water is applicable to wine?! Discussion may be aligned with wine only. If water standard applicable to wine, the other pesticides limit in water also applicable to wine. Please consider this

3.4. Food safety:

- Only comparing the exposure data with ADI the food safety of the wine cannot be compared. A detailed dietary risk assessment of Belgian population based on dietary intake of other food commodities and their MRL status with respect to detected pesticide only will give a risk assessment since the residue can enter in to body through other food also. This should be considered while concluding the risk assessment. The limitation should be clearly specified.

4 Conclusion: Considering the comment in 3.4; the conclusion may be modified with the limitation of the study.

******************************
