# Supplementary material for: The temporal variation in pesticide concentrations within matured French wines
Source: PLoS One. 2025 Feb 11;20(2):e0317086. doi: 10.1371/journal.pone.0317086 (PMC11813125; doi:10.1371/journal.pone.0317086)
Supplement: Attachment — Submitted filename: Manuscript Number PONE-D-24-17146R1.docx [file pone.0317086.s014.docx]

Manuscript Number PONE-D-24-17146R1

The temporal variation in pesticide concentrations within matured French wines.

**General comments from reviewer:**

It is a g good idea to screen bottled wine for pesticide residues. The range of pesticides analyzed is fair, which adds to the conclusions that can be drawn (although I don’t have a very good overview of pesticides approved in grapes), but the pesticide list need to be clarified better (e.g. in Appendix).

It can be confusing when you refer to «active substance» in figures, but use «PPP» in the text. The «PPP» should in most cases be understood as «active substance in PPP». A PPP is the formulated product, that contains one or more active substances and co-formulants. You refer to [12] Sykalia et al (2024), and they use the term «Pesticide» for all the active substances in their paper – as is also commonly used in other papers. Although «pesticide» is also technically defined as the formulated product, I suggest you use «Pesticide» in stead of «active substance» and «active substance in PPP», since this use of the term is so common. You may clarify this use of the phrase pesticide in the introduction. This will also harmonize with your Title, where you use «Pesticide». (Piperonyl butoxide, being a synergist and not a pesticide, may need some remarks). If you decide to keep «active substances» as a term, the use of «PPP» in the text need to be critically evaluated to see if it is used as per definition.

Line 20, 93, 227, 261, 502: How many active substances were detected in the samples. Different things are stated (20, 21 or 23 substances).

Please check the wording of every Figure text/Table text (headings), there are many non-precise headings. E.g. Table A1: «List French wines tested», which should be «A list of the French wines tested».

The paper puts weight on findings above LOD and findings above LOQ, hence it is necessary to know the respective LOD and LOQs and this needs to be clarified better (for GC-ECD analysis).

The language and clarity in the sentences can be improved. See separate pages at the end of this document, with some of my *suggested* amendments in blue text.

**Minor aspects in Introduction:**

Line 78: please remove «celery leaves, and majoram»

Line 83-84: it is unclear what is meant with this sentence. Please insert an update.

**Minor and major aspects in Material & Methods:**

Please update in Figure 1:

- The Legend text «The distribution of the 84 wine bottles by year of production and region of production in France».
- The dark blue and the blue columns are quite similar in colour. Can the dark blue colur be edited to another colour?

Line 120-121: please remove «The cartridge effectively retained all PPPs present in the wine», as this is not known – the wine could contain other pesticides not analyzed for.

Line 121-122: was the cartridge rinsed with hexane and then eluted with AcN? It seems later in the text that the cartridge was eluted with 2 ml hexane and 8 ml acetonitrile. Hence the hexane is not a rinse, but use as an eluent? Please insert a clarification.

Line 129, 132 a.o.: Please write «wine sediment» in stead of only «sediment», as many readers will associate «sediment» with sediments in streams and rivers. This relates to several places in the text. Please insert updates.

Line 132-141. When was acetonitrile added to the wine sediment sample? Please insert.

Line 142: How was the evaporation performed. Please insert.

Line 157: Was matrix-matched calibration performed, or were standards solved in solvent only? Please insert description.

**2.2 LC-MS/MS Analysis:**

Line 166-167: triple quadrupole mass spectrometry (not double mass). I suppose triple quadrupole was used? Please specify the model of the LC-MS/MS.

Line 177: The LOQs for the LC-MS/MS method is stated below Table A.2, but the LOQs for the GC-ECD method is not stated enywhere. Please add. Please state LOQs in µg/L (not mg/L).

Line 182-183: The decription of how the quantification was performed is very unclear. Please rewrite. Was processed (worked-up) matrix calibration standards used for the quantification? Or were some pesticides quantified with solvent standards/matrix-matched standards and some pesticides with processed matrix calibration standards? Please specify in the text. May add the range of the calibration standards.

How many pesticides were targeted in the LC-MS/MS method? Please specify in the text.

Which of the detected pesticides in the wine were detected with the LC-MS/MS method. Please specify in the text.

What was the LO

Please add a list of all the pesticides in the analytical methods, either in 2.2 LC-MS/MS section (and 2.3 GC-ECD section) or in an Appendix.

**2.3 GC-ECD Analysis:**

Please specify the model of the GC-ECD.

Please add a description on how the quantification with calibration standards was performed. May add the range of the calibration standards.

Which of the detected pesticides in the wine were detected with the GC-ECD method. Please specify in the text.

**3 Results:**

Line 230: Explain why cadusafos was non-quantifiable (due to cadusafos levels being below the LOQ).

Line 249: It should be commented that carbendazim is also a metabolite of thiophanate-methyl and benomyl. Have any of these been approved in viticulture in France?

Line 297: This is a valid point (on systemic vs non-systemic/contact). However, you may add some comments on application rates and application dosages, which may (usually) be higher for fungicides than for insecticides? The fields being treated more often with fungicides than with insecticides, and at higher dosages for the fungicides?

Line 307: Explain the colours used in Table A.6. What/why has some values been considered «high levels» whereas other leves have been considered low? (e.g.

Line 333 (Figure 6), line 336, Figure 10 a.o.: It is better to use the phrase «production year of the wine» in stead of «the year of wine», «over the years». «Age of the wine» can be used.

Line 344: The analysis method(s) did not include analysis of obsolete and banned pesticides such as DDT and metabolites, aldrin and other POPs. Hence residues of these in the (old) wine may have been missed. Please insert a comment on this in the paper. You may also want to comment on metabolites not being included in the analysis method.

Line 398: A comment: the lower pesticide levels in the wine sediments as compared to the wine liquid phase – even though many of the fungicides exhibit high sorption coefficients - may also be due to the ethanol content in the wine, as the ethanol increases the pesticides’ solubility in the wine liquid.

Line 403: rewrite «which was not possible in wine». It is an unclear statement.

Line 423: The Table A.11 refers to the pesticides detected in one of the analyzed wine bottles, not all botles. Please correct.

Line 445: «Pesticide degradation adheres to a first-order kinetic model..». Pesticide degradation may also be bi-phasic, e.g. fast degradation of dissolved pesticide with a slow degradation of sorbed pesticide. It depends on the system/matrix. Please modify. (e.g. Pesticide degradation often adheres to a first-order kinetic model..».

Line 446: «This model implies that as pesticide levels diminish, the time required for further degradation extends» What do you mean here? In a single-first-order linear degradation (SFO), the degradation rate is constant throughout. Please remove, or rephrase.

Line 449: this is a good point.

Line 484: «Most pesticides are banned in grape production,..». Rather rephrase this sentence to e.g. «Pesticides not approved for use in grapes have default MRL values set at 0.01 mg/kg or 0.05 mg/kg.»

Line 485: MRLs in grapes are calculated into MRL in wine, using a processing factor, to account for pesticide residues in the final product (wine). There exists calculated EU MRLs in wine.

Line 488: «*Considering the quantity and variety of pesticides detected in the older wines, these products fail to meet current European regulatory standards and should normally be taken out of the market*». This needs more explanation and justification. You are not discussing the MRL levels with the levels detected in the individual wine samples here? (i.e. Table A6). Which samples exceeded the MRL levels? «The quantity of pesticides» means the number of pesticides detected, not their pesticide concentrations. The number/sum of pesticides are not regulated?

Actually, the pesticide levels in the wine samples are not really discussed in the paper. Table A.6 is presented but the levels are not really discussed in the Results and Discussion section? If you choose not to discuss the levels found compared to the calculated MRLs in wine, it is OK. But then you cannot make the statement of *Considering the quantity and variety of pesticides detected in the older wines, these products fail to meet current European regulatory standards and should normally be taken out of the market*».

Line 521-530: These are valid points. Though I lack some coherence (or better explanations) between the findings and the conclusions.

Table A.4:

Please state the mean pesticide concentration of positive samples as µg/L (not mg/L). (Since in Table 1.6. concentrations are stated in µg/L). Are the concentrations in table A.4 the sum of residues found in wine sediment and in wine liquid?

Please remove the pesticides that were not-quantifiable; the Table heading says the Table contains the pesticides in wine with concentrations higher than LOQ.

Make sure the mean concentrations are only for the positive samples (as stated), and that the mean does not include the zero samples? I note that many of the mean concentrations are below the LOQ of 1 µg/L for LC-MS/MS compounds?

Revised Manuscript with Track Changes

*Scientific article*	1

**The temporal variation in pesticide concentrations**	2

**within matured French wines.**	3

**Pieter Spanoghe^1^*, Jasmine De Rop^1^, Lilian Goeteyn^1^, Agnieszka Deja-Muylle^1^, Hannah**	4

**Vanderstappen^1^, Lotte Neckebroeck^1^, Dries Verhaegen^1^, Pauline van den Hove^1^, Joachim Nery^2^ and**	5

**Erik Meers^2^**	6

1. Department of Plants and Crops, Crop Protection Chemistry, Coupure Links 653, 9000 Gent, Belgium	7
2. Department of Green Chemistry & Technology, Resource Recovery in the Biobased Economy, Coupure Links 653, 9000 Gent, Belgium	8

***** Correspondence: author: Pieter.Spanoghe@UGent.be	9

**Abstract:** Numerous organizations worldwide are diligently working to regulate	10

the composition of food products, with a particular focus on pesticide content.	11

Each year, several substances are classified as hazardous to human health and	12

subsequently banned from agricultural use. In this study, we address the age-old	13

question: "Does wine improve with time?" from the context of pesticide	14

composition. We gathered wine samples from renowned French winemaking	15

regions, covering the years 1935 to 2000, to assess pesticide levels and identify	16

specific substances. Our objective was to determine if any currently banned	17

pesticides were present in these aged wines and whether the detected levels pose	18

health risks under typical daily consumption patterns. Our findings revealed the	19

presence of trace amounts of 20 different active substances, proceeding from Plant	20

Protection Products (PPPs), in most of the wine samples, albeit at levels considered	21

non-threatening to human health. Notably, one sample exhibited an alarmingly	22

high concentration of carbaryl, surpassing toxic consumption thresholds. This	23

study prompts discussions regarding the prioritization of pesticide testing in	24

various products and whether stringent regulations should be upheld in the wine	25

selling sector.	26

27

**Keywords:** pesticides; human exposure; wine; consumption; old wine testing	28

| **1 Introduction** | 29 | |  |
| --- | --- | --- | --- |
| The vine (*Vitis vinifera*) is an economically significant crop [1] renowned for its grape production. | 30 | |  |
| Grapes serve various purposes, not limited to fresh consumption or drying, but also encompass the vital | 31 | |  |
| art of winemaking. It is widely acknowledged that agriculture, including wine cultivation, employs a | 32 | |  |
| range of plant protection products (PPPs) such as insecticides, fungicides, and herbicides [2]. These are | 33 | |  |
| integral to safeguarding cultivated crops and ensuring bountiful harvests. Over the years, both in | 34 | |  |
| Europe and globally, there has been a growing awareness of the health and environmental implications | 35 | |  |
| associated with the extensive use of PPPs [3,4]. Consequently, new legislation has emerged, leading to | 36 | |  |
| increased restrictions on their usage (e.g. EU legislation regarding the bringing on the market of new | 37 | |  |
| PPP). This shift in policy is driven by concerns related to potential long-term effects on human health | 38 | |  |
| and the environment, as well as the enduring presence of some persistent PPP residues in our | 39 | |  |
| surroundings long after their application [5]. The aim is to strike a balance between agricultural | 40 | |  |
| productivity and the well-being of ecosystems and human populations. | 41 | |  |
| Before 1991, there was a lack of uniformity in legislation across European Union (EU) Member | 42 | |  |
| States regarding the use of PPPs. However, in 1991, the European Commission initiated a pivotal shift | 43 | |  |
| by introducing the first directive (Directive 91/414/CEE) aimed at regulating the authorization of active | 44 | |  |
| substances for use in European agriculture. In 2009, this legislation underwent a comprehensive | 45 | |  |
| revision, leading to the current regulation (AG) No 1107/2009, which remains in effect today [6,7]. This | 46 | |  |
| updated framework places a strong emphasis on the safe application of PPPs, ensuring their | 47 | |  |
| compatibility with all life forms on Earth, including humans and animals, while safeguarding the | 48 | |  |
| environment. It also introduces guidelines for the sustainable utilization of these products. Guidance | 49 | |  |
| sets what maximum residues of PPPs can be present in agricultural products. Each agricultural product | 50 | |  |
| is tested for the presence of PPP before consumption and that amount (residue) is compared with | 51 | |  |
| Europe's MRL (Maximum Residue Level) database. Approved active substances undergo periodic re- | 52 | |  |
| evaluation and may be either retained or banned from agricultural use, resulting in a dynamic landscape | 53 | |  |
| of authorized PPPs. Pesticides usage, safety, influence on environment and finally their content in food | 54 | |  |
| products are also important topics of The European Green Deal that sets crucial goals for climate till | 55 | |  |
| year 2050 [8]. | 56 | |  |
| Awareness of food safety, especially in alcoholic beverages, has increased significantly in recent | 57 | |  |
| years. Pesticide use in wine production is a major concern due to its ecological and health implications. | 58 | |  |
| Analytical methods for assessing pesticide levels primarily target grapes, with techniques such as high- | 59 | |  |
| performance liquid chromatography (HPLC) and gas and liquid chromatography (GC, LC) being | 60 | |  |
| commonly employed. Sample preparation methods vary and include QuEChERS, solid-phase | 61 | |  |
| microextraction (SPME), pressurized liquid extraction (PLE), dispersive liquid-liquid microextraction | 62 | |  |
| (dLLME), and accelerated dispersive liquid-liquid microextraction (ADLL-ME) [9]. These methods are | 63 | |  |
| also applied to analyze the final consumer product, including both the liquid and sediment phases of | 64 | |  |
| wine. For instance, [10] identified 48 pesticides in wine samples using liquid chromatography-tandem | 65 | |  |
| mass spectrometry (LC-MS/MS). [11] introduced a green solvent-based DLLME/HPLC-MS method for | 66 | |  |
| extracting and analyzing pesticide content. More recently, [12] validated QuEChERS extraction | 67 | |  |
| combined with Gas Chromatography-Mass Spectrometry (GC-MS) as the most optimized method for | 68 | |  |
| testing pesticide content in wine, applying their approach to both grape and wine phases. Research in | 69 | |  |
| this area extends beyond commercially available wines to include organic varieties, as evidenced by | 70 | |  |
| studies such as [13]. This comprehensive approach ensures that pesticide levels are accurately | 71 | |  |
| monitored throughout the production process, from grape cultivation to the final product. | 72 | |  |
| Maximum Residue Limits (MRLs) for pesticide active ingredients are established globally to ensure | 73 | |  |
| the safety of food consumption. According to a 2016 European Union report on pesticide residues, the | 74 | |  |
| incidence of MRL exceedance in wine is relatively low [14]. However, wine production begins at the | 75 | |  |
| vineyard, where the frequency of pesticide residues in untreated grapes is typically high. It's crucial to | 76 | |  |
| consider that processing factors also affect pesticide levels. Processed grapes, such as those used in | 77 | |  |
| making raisins, celery leaves, and marjoram, often show some of the highest frequencies of MRL | 78 | |  |
| exceedances among processed foods [15]. Therefore, when evaluating MRLs in wine, it's essential to | 79 | |  |
| account for processing factors. These factors not only reflect the impact of processing on the food | 80 | |  |
| product but also on the chemicals themselves. This underscores the importance of continuous | 81 | |  |
| monitoring and evaluation throughout the wine production process, from the grapes in the vineyard to the final bottled product. However, stored products often escape such evaluations, highlighting a gap in ensuring ongoing safety and compliance.  For consumers, savoring a glass of wine does not typically conjure thoughts of the pesticides used in vineyards. Although studies have been made to indicate the pesticide content in wines available currently on the market [13], trend in consumption is more focused on organic values than possible, health influencing, pesticide content [16]. Similarly, consumers may not consider the possibility of residual traces of older PPPs, which are no longer authorized for use on grapes, persisting in the wine over time. This research endeavors to investigate the presence of such traces in 84 French wines produced before the year 2000. This study not only examines the wine, which consumers drink, but also delves into the solid phase, including sediment and lees, left behind in the bottle. In addition to detection of 21 active substances/residues, the study assesses the presence of copper residues, a significant inorganic component renowned for its fungicidal properties and use in viticulture. Furthermore the risk of the detected levels of pesticides was assessed for different age groups and adults versus children. This allowed insight into toxicity due to causal and prolonged usage.   2 **Material and methods**   *2.1. Sampling and clean-up extraction of active substances from wine and sediment*  A total of 84 bottles of French red wines were acquired from wine collectors within the author's personal network, including family and friends. This study primarily focused on older wine production, spanning from the years 1937 to 2000, thereby covering a significant historical range. The sample set was thoughtfully selected to represent various wine-growing regions in France, and a detailed breakdown can be found in the appendix (Table A.1). Figure 1 illustrates the distribution of wine bottles by their age. | | 82  83  84  85  86  87  88  89  90  91  92  93  94  95  96  97  98  99  100  101  102  103  104  105  106 | |

107

0

2

4

6

8

10

12

14

16

18

20

1930-1969

1970-1979

1980-1989

1990-2000

Number of wine bottles

Roussillon

Loire

Bourgogne

Côte du Rhône

Bordeaux

other

| **Figure 1. The** distribution of the wine bottles by year of production and region of production in France.  Each of the wine samples underwent testing for the presence of active substances - residues stemming from PPPs. Each bottle provided two samples: one from wine and the other from the solid phase, comprising sediment and lees. The bottle contents were filtered to separate the wine from the solid components, including lees. This filtration process involved passing 400 to 500 mL of wine through filter paper (Whatman GF/D glass microfiber filters, 47mm diameter), leaving behind any residual matter.  The filtered wine liquid phase underwent solid-phase extraction, employing an efficient and robust | 108  109  110  111  112  113  114  115 |
| --- | --- |
| technique based on the method previously described by [17]. Sep-Pak® C18 Classic Cartridges (Whatman Glass | 116 |

| Microfiber Binder Free Filter, 2.7 Micron, 2.2 GF/D, 4.7cm Diameter) were employed in this process. The C18 cartridge was first activated with 2 mL of methanol HPLC grade (Chem-lab NV, 2.5L) and rinsed with 2 mL of HPLC water (Chem-lab NV, 2.5L). Subsequently, 50 mL of the filtered wine sample was diluted with 50 mL of Milli-Q water (in house dispenser) and passed through the cartridge. The cartridge effectively retained all PPPs present in the wine. Following this step, the cartridge was rinsed with 2 mL of HPLC grade hexane (Alltech Associates Inc, 2.5L) and 8 mL of HPLC grade acetonitrile (Chem-lab NV, 2.5L), which carried the analytes. This sample preparation method was adapted from the methodology outlined by [17]. It's worth noting that the dilution of wine samples was found to significantly impact extraction efficiency, with a 1:1 dilution ratio yielding superior results compared to undiluted wine samples. This effect is likely due to the high ethanol content typically found in wine.  The residual material on the filter paper was allowed to air-dry to determine accurate lees masses.  The QuEChERS method, which stands for 'Quick', 'Easy', 'Cheap', 'Effective', 'Rugged', and 'Safe', was employed to extract PPPs from the sediment. This method consists of two main steps: first, the extraction of desired analytes using acetonitrile, followed by the purification of the extract through Solid-Phase Extraction (SPE), as outlined by [18].  The dried sediment, along with the filter paper, was transferred to a Falcon tube (Nerbe Plus, PP, 50 ml), containing 1.5 g of sodium citrate (Sigma-Aldrich, 1kg), 1.5 g of sodium chloride (Sigma-  Aldrich, 1kg), 0.75 g of sodium hydride (Sigma-Aldrich, 1kg), and 6 g of magnesium sulfate (SigmaAldrich, 1kg). The salts are used as a buffer to maintain optimal pH during extraction in acetonitrile, while magnesium sulfate is used to remove water. The sample was vigorously shaken [by hand?] and centrifuged at 10,000 rpm for 5 minutes. Subsequently, 10 mL of the upper layer was pipetted off to a pre-prepared tube (DisQuE® 1200 mg, WATERS) containing MgSO4, PSA (Primary Secondary Amine, and C18. After thorough shaking, the PSA tubes were centrifuged at 10,000 rpm for 5 minutes. Subsequently, 5 mL of the purified sample was transferred to a flask for solvent exchange  [18].  To facilitate LC-MS/MS analysis, the extracted samples from both the wine sediment and the wine were evaporated to exchange solvents. The PPPs were redissolved in 2 mL of a solution comprising 90% Milli-Q H2O and 10% ACN (acetonitrile) for LC-MS/MS analysis. The flask was briefly placed in an ultrasonic bath (Elma® Transsonic T700) before x ml was transfered to vial. Vials were stored at X C awaiting analysis.  To assess the presence of old chlorinated compounds, a GC-ECD analytical method was employed. Two specific extraction methods were tested for filtered liquid wine spiked with a known concentration of pesticides, with the first employing C18 Sep-Paks. These packs were rinsed with 5 mL of a 50/50 mixture of methanol and water, followed by elution with 2 mL of hexane and 8 mL of ACN. The resulting outcome was redissolved in 2 mL of hexane after evaporation. As no pesticides were detected in the spiked wine with this procedure, a second extraction method utilizing the conventional QuEChERS method was tested. This involved combining 10 mL of filtered red wine with 15 mL of ACN, followed by vigorous shaking for 5 minutes. Subsequently, salts were added, and the mixture was shaken for an hour before centrifugation at 10,000 rpm for 5 minutes. Next, 10 mL was pipetted off and transferred to a PSA tube for cleanup, followed by another centrifugation step. Afterward, 8 mL was removed, evaporated, redissolved in 2 mL of hexane, and prepared for analysis. Standards used were of 99,9% purity, originating form Sigma Aldrich. Quantification was done by external standard calibration in solvent. For validation, matrix effect was evaluated using spiked matrix samples and spiked blank matrix extracts.  To determine the presence and quality of copper content in wine samples, the wine matrix was subjected to a destructive process as per the procedure specified by Vito: WAC/III/B/001. This involved adding 4 mL of HNO3 to 30 mL of wine in a 100 mL beaker, which was then covered with a watch glass. The destruction process occurred over 2 hours at 120°C and was quantitatively transferred to a 50 mL volumetric flask as outlined by [19].  *2.2. LC-MS/MS Analysis*  Following sample collection and extraction, the wines were subjected to liquid chromatography analysis coupled with triple quadrupole double mass spectrometry, using Model X LC-MS/MS and | 117  118  119  120  121  122  123  124  125  126  127  128  129  130  131  132  133  134  135  136  137  138  139  140  141  142  143  144  145  146  147  148  149  150  151  152  153  154  155  156  157  158  159  160  161  162  163  164  165  166  167  168 |
| --- | --- |
| an Acquity UPLC BEH C18 1.7µm column (2.1*100mm, Waters). The column temperature was maintained at 40°C, while the desolvation temperature reached 450°C. The mobile phase consisted of acetonitrile (solvent B) and water with 0.1% | 169 |

| formic acid (Merck) (solvent C), flowing at a rate of 0.4 ml/min. Solvent B and C were distributed in varying proportions between 1% and 99% over time. Each run had a duration of 11 minutes.  It's worth noting that during the analysis, some active substances from the wine samples may not have been completely extracted or could have been lost. Interactions between different active substances and other extracted components might have led to a matrix effect, as documented by [21]. To account for these variations and their impact on the detected concentrations, we determined the recoveries of the PPPs. The recovery values for the active ingredients in both wine and sediment, along with validation parameters like Limit of Detection (LOD) and Limit of Quantification (LOQ), can be consulted in Table A.2 and Table A.3 in the appendix.  The Limits of Detection (LOD) and Limits of Quantification (LOQ) were determined by injecting the lowest concentration eight times and by analyzing the signal-to-noise (S/N) ratio. The variability was assessed by repeating the measurements three times for the LOD and ten times for the LOQ. The quantification has been according to the standard curve. Quantification was performed based on pre and post spiking of the wine (pre-spiking of the wine, post – spiking of the wine extract). The fixed value used is based on the lowest standard concentration injected.  *2.3. GC-ECD analysis*  For GC-ECD analysis, a capillary column (HP5MS, 30.0m250mm0.25µm) was employed. The inlet pressure was set at 93 bar, with a temperature of 200°C, resulting in a flow rate of 62.5 ml/min. The detector was heated to 250°C. The temperature program for the column initiated at 60°C, increased to 150°C at a rate of 20°C/min, further increased to 250°C at a rate of 15°C/min, held constant for 2 minutes, increased to 270°C at a rate of 30°C/min, maintained for 10 minutes, and ultimately reached 280°C at a rate of 30°C/min, holding steady for 11 minutes. The entire runtime for this analysis was approximately 35 minutes.  *2.4. Copper content determination*  To determine copper content in the wine samples, we utilized ICP-OES (Inductively Coupled Plasma Atomic Emission Spectroscopy), employing a Varian Vista MPX + autosampler in accordance with Vito: WAC/III/B/010 [22].  2.5. Food safety assesment  To determine whether a food safety issue is present when drinking aged wines, a risk analysis was performed. This involved a comparison of a daily intake of wine with the acceptable daily intake (ADI) of each substance, which represents the maximum daily intake of a substance that a person may consume over a long period without incurring health risks. The ADI is expressed in mg per kg body weight per day (mg/(kg BW*d)) and can be found for each active substance in an appendix in Table A.4. It refers to chronic toxicity or long-term exposure [23].  In subsequent calculations, only the liquid part of the wine was considered, with the assumption that consumers do not ingest the sediment of the wine. According to the latest data from the European Food Safety Agency, the average wine consumption in Belgium is reported to be 300g/day, while frequent wine consumers reach 653g/day [24]. Calculations here presented are based on the assumption that the mass density of wine is equivalent to that of water, i.e., 1 kg/m³. Belgian men are 1.76 m tall on average and weigh 79 kg. The average height for Belgian women is 1.64 m and the average weight is about 66.7 kg [25]. With these data, consumption per kg body weight was calculated according to the | 170  171  172  173  174  175  176  177  178  179  180  181  182  183  184  185  186  187  188  189  190  191  192  193  194  195  196  197  198  199  200  201  202  203  204  205  206  207  208  209  210  211  212  213  214  215 |
| --- | --- |
| formula below. | 216  217 |

218

219

220

221

| 𝑏 = ♀ 66.7 𝑜𝑟 ♂ 79 kg BW   3 **Results and discussion**   3.1. *PPP residues in the wine liquid phase*   3.1.1. Types of PPPs detected  Among the 21 active substances detected, 20 are quantifiable, as depicted in Figure 2. Carbendazim emerges as the most prevalent active substance, being present in 88% of the samples, corresponding to 74 wine samples. Following closely is cadusafos, detected in 60% of the samples (50 wine samples). It's worth noting that unlike carbendazim, all cadusafos determinations are non-quantifiable. Piperonylbutoxide and metalaxyl were also notable findings, appearing in 43% and 40% of the samples, respectively, amounting to 36 and 34 wine samples, respectively. For detailed information on the detected active substances, including their mean concentrations above the LOQ, please refer to Table A.5 provided in the appendix.  According to [26], several of the active substances detected in our study, namely azoxystrobin, difenoconazole, prochloraz, and tebuconazole, are primarily employed for the control of powdery mildew in Belgium. Azoxystrobin and difenoconazole also find application in addressing downy mildew, with additional support from metalaxyl, dimetomorph, and copper. Furthermore, these two substances are utilized in the control of grey mold. Pyrimethanil, tebuconazole, and diethofencarb can also be harnessed to combat this particular mold. When it comes to battling black rot, azoxystrobin, difenoconazole, tebuconazole, and metalaxyl are the go-to choices. Examining Figure 2, it becomes evident that a significant portion of the identified Plant Protection Products (PPPs) can be attributed to their necessity in safeguarding crops against downy mildew, powdery mildew, black rot, and grey mold. However, the presence of certain substances, such as carbendazim, cadusafos, and carbaryl, in many samples raises questions since these substances are no longer approved for use on grapes by the European Commission, aligning with current legislation. Notably, carbendazim, according to the study by [27], exhibited remarkable efficacy in reducing powdery mildew incidence by 75% in mangoes. It also displayed effectiveness in combating rot and downy mildew (APVMA, 2007). Despite its wide-ranging antifungal properties, carbendazim is no longer authorized due to its adverse impact on human health [28]. Given its broad-spectrum activity, it is reasonable to assume that this substance was frequently applied to protect *V. vinifera*. In contrast, cadusafos and carbaryl serve as insecticides rather than fungicides. They target nematodes, aphids, and butterflies, | 222  223  224 225 226  227  228  229  230  231  232  233  234  235  236  237  238  239  240  241  242  243  244  245  246  247  248  249  250  251  252 |
| --- | --- |
| respectively. Furthermore, piperonyl butoxide, detected frequently, acts as a synergist for (organic) insecticides, indirectly contributing to the reduction of pest insect populations [29]. | 253  254 |

255

2

5

50

2

1

0

3

9

9

4

0

3

9

27

0

4

5

7

5

4

0

3

7

0

18

73

3

12

2

18

7

1

6

25

9

1

2

17

8

7

5

10

0

10

20

30

40

50

60

70

80

Azoxystrobine

Benalaxyl

Cadusafos

Carbaryl

Carbendazim

Chlorpropham

Diethofencarb

Difenoconazole

Dimethomorph

Diuron

Fenbuconazole

Hexaconazole

Metalaxyl

Piperonylbutoxide

Prochloraz

Propanil

Pyrimethanil

Tebuconazole

Tebufenozide

Triademifon

Triademinol

**Numer of detections**

**Detection of active substance in wine (N=84)**

**<**

**LOQ and >LOD**

**>**

**LOQ**

| **Figure 2.** Number of times each active substance was detected in wine. In the analyzed wine bottles, with the | 256 |
| --- | --- |
| distribution according to detectable (<LOQ and >LOD) and quantifiable (>LOQ). | 257  258 |
| Plant protection products are categorized into several groups, with the primary ones being | 259 |
| fungicides, insecticides, and herbicides. In the scope of this study, the majority of the identified active | 260 |
| substances exhibit fungicidal activity, totaling 14 out of the 23 active substances. Additionally, we | 261 |
| detected five types of insecticides, three types of herbicides, and one synergist that enhances the | 262 |
| effectiveness of insecticides (please refer to Table A.5 in the appendix for details). Figure 3 provides a | 263 |
| breakdown of the identified active substances based on their respective types. Fungicides emerge as the | 264 |
| predominant category, with 245 detections recorded across the 84 wine samples. In contrast, the second- | 265 |
| highest number of detections pertains to insecticides, accounting for 82 observations. Notably, the color | 266 |
| distribution of the bars in Figure 3 reveals an interesting trend. Fungicides tend to occur primarily in | 267 |
| quantifiable concentrations, constituting 77% of the total detections. Conversely, insecticides are more | 268 |
| frequently found in lower concentrations, falling either below the LOQ or above the LOD, making up | 269 |
| 63% of the detections in this range. | 270 |

271

| **Figure 3.** Distribution of active substances in the analyzed wine bottles according to their type with the distinction | 272 |
| --- | --- |
| between concentrations to be quantified (> LOQ) and the detectable ones (< LOQ and > LOD). | 273 |
| The prevalence of fungicides compared to other types of Plant Protection Products (PPPs) can be | 274 |
| logically explained. Fungicides exert their effects systemically, targeting fungi present on or within the | 275 |
| grapevine and its fruit. Moreover, *Vitis vinifera*, the grapevine species in focus, is notably more | 276 |
| susceptible to fungal diseases compared to insect pests. In essence, fungicides are indispensable for | 277 |
| managing fungal threats, as they act throughout the grapevine's entire structure, safeguarding both the | 278 |
| plant and its fruit. In contrast, insecticides primarily address insect pests that often reside on the plant. | 279 |
| Consequently, their action is primarily directed at the surface of the grapes, as they aim to eliminate | 280 |
| insects without extensive systemic activity within the plant. Herbicides, on the other hand, are relatively | 281 |
| less common in our findings. This can be attributed to their mode of action, which involves targeting | 282 |
| and being applied to the plants situated between the vines rather than directly impacting the grapes | 283 |
| themselves. The herbicides detected in quantifiable quantities (comprising 60% of the total herbicide | 284 |
| detections) typically exhibit systemic properties, as they are absorbed through the root system of the | 285 |
| vines and subsequently transported into the grapes. | 286 |
| Pesticides can be categorized into systemic and contact pesticides. Systemic pesticides are absorbed | 287 |
| by the plant and operate by transport and redistribution, while contact pesticides act at the specific site | 288 |
| where they have been applied. Figure 4 illustrates the total number of detections of systemic and contact | 289 |
| PPPs across the 84 analyses. It is evident that systemic PPPs are more frequently detected than contact | 290 |
| PPPs, in line with our expectations. Specifically, systemic PPPs were detected a total of 258 times, while | 291 |
| contact PPPs were found only 89 times. Among the systemic PPPs, 76% of the total number of detected | 292 |
| observations were quantifiable (>LOQ), whereas for the contact PPPs, only 31% were present at | 293 |
| concentrations above the LOQ. This demonstrates that systemic PPPs are generally present in relatively | 294 |
| higher concentrations compared to contact agents. This difference can be attributed to the fact that | 295 |
| systemic PPPs penetrate the plant's cuticle and migrate into the plant tissue (such as the grape pulp), | 296 |
| whereas contact PPPs are less prevalent in wines due to their susceptibility to being washed off by rain | 297 |
| or runoff from leaves and grapes. Consequently, contact PPPs are often no longer present at the time of | 298 |
| harvest, whereas systemic PPPs persist for a longer duration. It is worth noting that when wines mature | 299 |
| in typical oak barrels, there is a possibility that PPPs from the wood of the barrels may permeate the | 300 |
| wine, and vice versa. Consequently, an exchange of active substances may occur between different | 301 |
| wines stored in previously used barrels. | 302 |

303

| **Figure 4.** Distribution of active substances in the analyzed wine bottles according to their activity with the | 304 |
| --- | --- |
| distinction between quantifiable (> LOQ) and detectable (< LOQ and > LOD) concentrations. | 305 |
| 3.1.2. Number of PPPs detected | 306 |
| The results of residue analysis in wine are presented in Table A.6 of the appendix. Out of the 84 | 307 |
| wine samples analyzed, traces of 23 different active substances were detected, with concentrations | 308 |
| exceeding the LOD in at least one sample. Except for seven wine samples, the majority of wine samples | 309 |
| had at least one active substance detected (>LOD). For residues above LOD, a distinction was made | 310 |
| between detectable and quantifiable concentrations. | 311 |
| Across all wines, an average of 1.8 active substances were found in detectable concentrations (but | 312 |
| below LOQ), while 2.8 quantifiable substances were identified (as shown in Table A.8 in the appendix). | 313 |
| On average, a total of 4.6 active substances were identified per bottle. Figure 5 provides a comprehensive | 314 |
| representation of the number of active substances determined per bottle. | 315  316 |

317

**Figure 5.** Amount of active substances found in analyzed wine, per wine bottle (N=84).	318

| In 4 bottles (5%), exactly one active substance was detected. In 57 bottles (68%), between 2 and 5 | 319 |
| --- | --- |
| active substances were found. In 18 bottles (21%), more than 6 up to 10 active substances were present, | 320 |
| and 5 bottles (6%) even had more than 10 different active substances detected. Notably, six bottles | 321 |
| contained traces of 10 or more substances. It is worth mentioning that these six bottles with traces of | 322 |
| more than 10 active substances were all from the years 1995, 1996, 1997, 1998, and 2000. Within the scope | 323 |
| of our study, these wines are considered relatively young. | 324 |
| Figure 6 represents the relationship between age and the average number of active substances | 325 |
| detected using a point cloud. In this figure, the 2 wines from 1937 containing piperonylbutoxide are | 326 |
| represented by two points that lie far outside the point cloud. As extreme values, they leverage the trend | 327 |
| line and influence the result excessively. As can be observed from the graph there is a clear upward | 328 |
| trend between the number of active substances detected per bottle and the year of the wine. The younger | 329 |
| the wine, the more traces of active substances are found in the bottle. | 330  331 |

332

| **Figure 6.** Relationship between the year of analyzed wine and the number of detectable active substances in the | 333 |
| --- | --- |
| wine. | 334 |
| Several assumptions can be made regarding the apparent increase in the number of active | 335 |
| substances found in wine over the years. The most apparent explanation is that there was, in fact, less | 336 |
| use of PPPs in the earlier years. However, there are other possibilities to consider. For instance, the shift | 337 |
| from broad-spectrum PPPs to selective ones since the 1990s. It became evident that broad-spectrum | 338 |
| PPPs also affected non-target organisms. Consequently, research began to focus on selective PPPs, | 339 |
| aiming to preserve beneficial control agents. The consequence of this trend was that more PPPs were | 340 |
| required to achieve the same level of crop protection. Similarly, a noticeable increased in the research | 341 |
| field of pesticides has prompted their increased usage in food production. | 342 |
| Another explanation could be that older wines contain PPPs that require a different single- | 343 |
| component detection technique, which might not have been detected using the extraction and detection | 344 |
| methodology applied in this study. Older bottles may also be in a more advanced state of degradation, | 345 |
| potentially influenced by lower alcohol content. It's important to note that there is a considerable time | 346 |
| gap between the youngest and oldest wines studied. The varying half-life degradation times of different | 347 |
| PPPs might have influenced the persistence of PPP residues in the bottles. | 348 |

349

| 3.1.3. PPPs detected in analyzed wines according to region | 350 |
| --- | --- |
| Table A.1 in the appendix provides information about the grape varieties used in conjunction with | 351 |
| specific wines. This data illustrates that certain grape varieties are frequently associated with particular | 352 |
| wine regions. For example, Pinot Noir is consistently found in the Burgundy region, while Cabernet | 353 |
| Franc, Merlot, and Cabernet Sauvignon are commonly linked to the Bordeaux region. However, it is | 354 |
| evident that these grape varieties are not exclusively limited to one region, and there is overlap between | 355 |
| them. For instance, the Cabernet Franc variety is also used in the Loire region. Figure 7 illustrates that | 356 |
| the average number of active substances per bottle of wine does not exhibit significant differences | 357 |
| among various wine regions in France. Therefore, no further conclusions can be drawn based on region | 358 |
| in relation to the use of PPPs. | 359  360 |

361

|  | 362 |
| --- | --- |
| **Figure 7.** Average number of active substances detected per bottle, broken down by region. | 363 |
| 3.1.4. PPPs detected in analyzed wines according to authorization | 364 |
| A noteworthy observation regarding the presence of PPPs in the wine, is the differentiation | 365 |
| between active substances that are still approved and those that are no longer approved by the European | 366 |
| Commission (EC) as of 2022. Out of the 23 detected PPPs, 13 are no longer approved, while 7 agents | 367 |
| remain approved for use in vineyards. Figure 8 highlights that there are still a considerable number of | 368 |
| plant protection products in wines that have not received approval from the EC. On average, each bottle | 369 |
| of wine contains traces of 2.9 non-approved agents compared to traces of 1.5 approved agents. These | 370 |
| findings are intriguing, as they indicate a higher presence of unapproved active substances in older | 371 |
| wines. It is important, however, to note that these results are specific to the analyzed wines. | 372 |
| Additionally, at the time of production, many pesticides were not restricted in the market. Moreover, | 373 |
| the concentrations at which these substances are detected (>LOQ or >LOQ and >LOD) do not differ | 374 |
| significantly between approved and unapproved agents. These results raise questions about whether | 375 |
| old wines containing unauthorized products are still permitted for sale in the market or if there are | 376 |
| exemptions for this type of food product. | 377 |

‘378

| **Figure 8.** Distribution of the average number of active substances per bottle for analyzed wines, according to their | 379 |
| --- | --- |
| approval by the European Commission in 2022. | 380 |
| *3.2. PPPs detected in wine sediment* | 381 |
| The results of the analyses of the sediment for each bottle of wine can be found in Table A.7 of the | 382 |
| appendix. When enjoying wine, it can be assumed, for the most part, that the wine is not shaken, and | 383 |
| only the liquid part is consumed, leaving the sediment in the glass or bottle. | 384 |
| A significant difference is observed when comparing the number of active ingredients in the | 385 |
| sediment per bottle to the wine phase. In the sediment, notably fewer active substances per bottle were | 386 |
| detected, with a maximum of only 5 active substances per bottle, while in the wine, this number reached | 387 |
| as high as 12. As indicated in Table A.9 in the appendix, in the sediment, an average of 0.8 active | 388 |
| substances were detectable below the LOQ, and an average of 1 active substance could be quantified | 389 |
| (>LOQ). Consequently, on average, there were 1.7 active substances present per bottle in the sediment. | 390 |
| Interestingly, in 15% of the sediment samples, no active substances were detected, whereas in each wine | 391 |
| sample, at least 1 active substance was present. In contrast to the wine, there was no clear correlation | 392 |
| between the year of the bottle and the number of active ingredients present per bottle in the sediment. | 393 |
| The relationship between the year and the number of active ingredients per bottle in the sediment | 394 |
| remained relatively consistent over the years (as shown in Figure 10). | 395 |

# Number of active substances detected per bottle in the sediment

**15**

**%**

**77**

**%**

**7**

**%**

**0**

**between 1 and 3**

**between 4 and 5**

396 **Figure 9.** Number of active substances detected in wine, in the sediment, per bottle of wine (N=84).	397

| Interestingly, only 12 active substances are detected in the sediment phase, while 20 substances are | 398 |
| --- | --- |
| detected in the wine. Out of the 12 detected substances in the sediment, 11 were found at concentrations | 399 |
| above the LOQ. Atrazine appears to be the only substance that is exclusively present in the sediment | 400 |
| and not in the wine itself. Notably, the two most frequently detected substances in both, wine and | 401 |
| sediment, were carbendazim and cadusafos. It's worth mentioning that cadusafos could be quantified | 402 |
| nine times in the sediment, which was not possible in the wine, in the liquid part. This suggests a | 403 |
| preference for cadusafos to be present in the sediment rather than the wine itself. | 404 |
| When comparing the average quantifiable concentrations between the sediment and wine, it was | 405 |
| observed that concentrations of the PPPs in the sediment were generally much higher. This is because | 406 |
| non-polar active substances exhibit hydrophobic characteristics and therefore have a lower affinity for | 407 |
| the liquid. | 408  409 |

410

| **Figure 10.** Relationship between the year of wine and the number of detectable active substances in the sediment.  *3.3. Copper in old wines*  Copper has historically been, and in some cases continues to be, employed in vineyards to combat fungal diseases. Excessive copper intake can lead to potential health problems. The World Health Organization (WHO), in conjunction with the European Union (EU), has established the maximum permissible standard for copper in drinking water at 2 mg/L [30]. Maximum residue limit (MRL) value for wine is 1 mg of Cu per L (EEC regulation No. 606/2009, Annex 1 A). Table A.10 in the appendix presents the results of copper analysis for the same samples from the 84 wines. It is worth noting that the copper standard was not exceeded in all samples, with the exception of three. The highest recorded value, twice above the norm, was 2.080 mg/L for sample 81. Two other samples contained just over of the allowed amount, measuring 1.308 mg/L and 1.154 mg/L for samples 83 and 84, respectively.  *3.4. Food safety*  Table A.11 in the appendix summarizes the maximum residue values found in the analyzed wine bottles for 20 active substances, with no traces of cadusafos detected. Exposure was calculated for both female and male consumers, based on their frequency of wine consumption. This approach provides a high-risk estimate for the presence of active ingredients in analyzed wines.  From these findings, it can be concluded that the Acceptable Daily Intake (ADI) was not exceeded for any active substances, except carbaryl. A noteworthy result was observed in sample F29, a 1969 Bordeaux known as Château Bries-Caillou (refer to Table A.1 in the appendix). Carbaryl | 411  412  413  414  415  416  417  418  419  420  421  422  423  424  425  426  427  428  429 |
| --- | --- |
| concentration exhibited an exceptionally high residue of 2.95 mg per 1L of wine (see Table A.11 in the appendix). The | 430 |

| exposure calculation for female drinkers in Belgium resulted in values of 0.013 and 0.029 mg per kg of | 431 |
| --- | --- |
| body weight per day for average and frequent consumption, respectively. These values significantly | 432 |
| surpass the ADI for carbaryl, which is 0.0075 mg/(kgBWd). Overall, the low ADI value indicates the | 433 |
| presence of more toxic substances, suggesting that a pesticide was present in wine samples at levels | 434 |
| exceeding safe toxicity thresholds by 77% and 285%. A similar calculation for male average and frequent | 435 |
| drinkers yielded values of 0.011 and 0.024 mg/(kgBWd), respectively, also exceeding the ADI for | 436 |
| carbaryl by 49% and 225%. Table A.11 also compares female and male exposure to the Acute Reference | 437 |
| Dose (ARFD), revealing that carbaryl toxicity was significantly exceeded for both genders, regardless of | 438 |
| their wine consumption frequency. | 439 |
| The most stringent ADI (0.0004 mg/(kgBWd)) is established for cadusafos. Residues of cadusafos | 440 |
| were found in 34 wine samples, but none exceeded the LOQ, making quantification impossible. | 441 |
| However, it can be deduced that a concentration of 0.1825 mg/L of wine would be necessary to achieve | 442 |
| an exposure of 0.0004 mg/(kgBWd). This concentration of 0.0004 mg is still well above the LOQ of 7.51E- | 443 |
| 5 mg/L of wine. As no cadusafos residue exceeded the LOQ, no safety concerns were raised. | 444 |
| Pesticide degradation adheres to a first-order kinetic model, characterized by an exponential | 445 |
| decrease in concentration over time. This model implies that as pesticide levels diminish, the time | 446 |
| required for further degradation extends. A notable finding from our study is that, thanks to the high | 447 |
| sensitivity of our analytical techniques, these pesticides can be detected even after a minimum of 20 | 448 |
| years of storage. Moreover, the presence of alcohol plays an important role in inhibiting microbial | 449 |
| activity, which would otherwise accelerate degradation. Consequently, pesticides dissolved in the | 450 |
| alcohol phase exhibit greater stability and persistence compared to those present in water. This results | 451 |
| in pesticides being more effectively preserved in alcoholic beverages than in other types of food and | 452 |
| drink. | 453  454 |
| This risk assessment does not take into account the cumulative exposure to pesticides. The risks | 455 |
| associated with pesticide residues in food are typically evaluated on a substance-by-substance basis in | 456 |
| wine. However, many pesticides exhibit similar effects, and their combined impact on human health | 457 |
| may be greater than when considered individually. Additionally, consumers do not limit their intake to | 458 |
| wine alone; residues of the same pesticides may also be present in other food items consumed | 459 |
| throughout the day. Therefore, a more comprehensive assessment of cumulative exposure is necessary | 460 |
| to better understand the potential health risks associated with pesticide residues in food. | 461  462 |
| In Belgium, the average wine consumption stands at 300g per day, while frequent wine consumers | 463 |
| consume 653g per day [24]. Consequently, the average Belgian ingests approximately 29.6g of ethanol | 464 |
| daily, while frequent consumers ingest more than double this amount, equating to 64.41g of ethanol per | 465 |
| day. Considering the grams per Body Weight (BW) statistics, Belgians consume 10g of wine per unit of | 466 |
| body weight daily, translating to 1g of ethanol per kg of body weight ingested. It is well-established that | 467 |
| chronic ethanol intake can lead to various diseases that subsequently impact lifespan. The most common | 468 |
| consequence of excessive ethanol consumption, known as alcohol intoxication, is liver problems, | 469 |
| alongside other metabolic issues [31]. Thus, when consuming wine, the primary health risks are more | 470 |
| likely to arise from ethanol presence rather than pesticide contamination. | 471  472 |
| The generally accepted 'safe' copper concentration in wine is less than 0.3–0.5 µg ml−1 [32]. While | 473 |
| many studies indicate that moderate wine consumption does not pose significant health risks related to | 474 |
| copper [33, 34], but all these findings are based on average wine exposure. Increased wine consumption, | 475 |
| particularly among wine connoisseurs or collectors of old wines, may present toxicological risks due to | 476 |
| limited public awareness about potentially banned ingredients. Prolonged consumption of copper in | 477 |
| wine has been associated with various health issues, including respiratory diseases, acute gastritis, and | 478 |
| decreased antioxidant capacity [35]. Therefore, it is essential to expand consumer knowledge not only | 479 |
| about pesticide residues but also about copper content in wine. | 480  481 |
| Maximum Residue Limit (MRL) legislation varies by region, with distinct MRL values established for | 482 |
| different pesticides. Importantly, these limits are typically set for grapes rather than the final wine | 483 |
| product. Most pesticides are banned in grape production, and default MRL values are commonly set at | 484 |
| 0.01 mg/kg or 0.05 mg/kg. MRLs in grapes are calculated into MRL in wine, using a processing factor, to account for pesticide residues in the final product (wine). We refer for details on the MRLs of wine grapes to the EC official website [36].  Considering the quantity and variety of pesticides detected in the older wines, these products fail to meet current European regulatory standards and should normally be taken out of the market. This raises a compelling question: why do consumers perceive and approach their exposure to pesticides differently when consuming aged wines compared to other agricultural commodities? In the latter case, products are frequently verified and removed from the market based on changes in legislation regarding pesticide content following novel findings on active substances when consumed or present in the environment. Consequently, aged wines might be better classified as collector's items due to their pesticide content rather than as everyday beverages. This intriguing contrast in consumer behavior and perception prompts further examination. It initiates a discussion about the factors influencing consumer attitudes and choices concerning pesticide exposure in various contexts. It also underscores the need for a deeper understanding of these dynamics.  **4 Conclusion**  The study's findings shed light on the persistent presence of unauthorized substances, as well as copper residues, in aged wines. Despite not focusing on cumulative pesticide exposure, it uncovered trace amounts of 20 different active substances derived from Plant Protection Products (PPPs)[7]. These substances, though found in small concentrations, underscore a broader issue of contamination in vintage wines. While most of the residue levels identified in the samples were deemed non-threatening to human health, one sample revealed an alarmingly high concentration of carbaryl, a pesticide that exceeded toxic consumption thresholds. This finding raises concerns about potential health risks, particularly in aged wines, which are often considered safe due to their long shelf life and perceived exclusivity.  The presence of such contaminants highlights the need for more rigorous pesticide testing  across a wide array of products, including those that, like vintage wines, are often overlooked due to their status as collectibles or luxury items. Currently, stringent food safety regulations are in place for modern products, but aged wines and other historical consumables often slip through regulatory cracks. This study underscores the necessity of extending contemporary testing protocols to these older items, ensuring that even vintage wines, which may have been produced under different regulatory frameworks, are subject to the same safety scrutiny.  A key point of this research is that it does not seek to address whether the pesticides found in  the analyzed wines were legal at the time they were produced. Instead, the primary focus is on the need to re-evaluate these older products against today's stringent food safety standards. As regulations evolve, substances that were once widely used in agriculture may now be banned, leading to potential risks from historical contamination.  This study aims to raise public awareness about the potential for pesticide residues in aged  consumables, particularly vintage wines that may have been stored for decades. As these products are often viewed as luxury goods or collectibles, consumers may not consider the possibility of contamination, assuming they are safe to drink. The research offers a new perspective on how these items should be assessed, suggesting that they be held to the same safety standards as other food and drink products currently on the market. This approach could ensure greater consistency in food safety across the board and prompt a rethinking of how aged or historically produced items are regulated. By bringing attention to this overlooked issue, the study encourages further discussion on how to handle the sale and consumption of such products, especially in light of modern food safety regulations that | 485  486  487  488  489  490  491  492  493  494  495  496  497  498  499  500  501  502  503  504  505  506  507  508  509  510  511  512  513  514  515  516  517  518  519  520  521  522  523  524  525  526  527  528  529 |
| no longer permit substances once considered acceptable. | 530 |

531

532

| **Authors contributions:** Supervision, writing – review and editing, methodology, | 533 |
| --- | --- |
| project administration, P.S.; investigation, L.G., J.d.R., J.N.; supervision, writing – | 534 |
| review and editing, E.M.; investigation, formal analysis, writing – original draft, | 535 |
| H.V., L.N., D.V., P.v.d.H.; writing - review and editing, ADM. All authors have | 536 |
| read and agreed to the published version of the manuscript. | 537 |
| **Funding:** This research received no external funding. | 538 |
| **Declaration of competing interest**: The authors declare that they have no known | 539 |
| competing financial interests or personal relationships that could have appeared | 540 |
| to influence the work reported in this paper. | 541 |
| **Data availability:** Data are contained within the article. | 542 |
| **Aknowledgments:** This work was supported by Gent University, Belgium. | 543 |
| **Conflicts of Interest:** The authors declare no conflict of interest. | 544 |

545
